# Exponentially Weighted Multivariate HAR Model with Applications in the Stock Market

**DOI:** 10.3390/e24070937

**Published:** 2022-07-06

**Authors:** Won-Tak Hong, Eunju Hwang

**Affiliations:** 1 Department of International Studies, Kyung Hee University, Yongin-si 17104, Korea; wontak@khu.ac.kr; 2 Department of Applied Statistics, Gachon University, Seongnam-si 13120, Korea

**Keywords:** multivariate HAR model, CUSUM test, exponentially decaying coefficients, stock price

## Abstract

This paper considers a multivariate time series model for stock prices in the stock market. A multivariate heterogeneous autoregressive (HAR) model is adopted with exponentially decaying coefficients. This model is not only suitable for multivariate data with strong cross-correlation and long memory, but also represents a common structure of the joint data in terms of decay rates. Tests are proposed to identify the existence of the decay rates in the multivariate HAR model. The null limiting distributions are established as the standard Brownian bridge and are proven by means of a modified martingale central limit theorem. Simulation studies are conducted to assess the performance of tests and estimates. Empirical analysis with joint datasets of U.S. stock prices illustrates that the proposed model outperforms the conventional HAR models via OLSE and LASSO with respect to residual errors.

## 1. Introduction

Financial market data are often correlated with each other and need to be analyzed together. If two or more financial data belong to the same category or reveal a similar pattern with strong correlation, the bivariate or multivariate datasets should be modeled simultaneously by an appropriate multivariate time series model to obtain good performance. Multivariate financial data with characteristics such as strong correlation and long memory have attracted much attention from econometricians and statisticians. Among various time series models, one of the most popular and powerful models capturing such financial features is the heterogenous autoregressive realized-volatility (HAR-RV) model [1]. Based on the HAR model, this paper considers a multivariate model to analyze joint time series data with strong cross-correlation.

The HAR-RV model has been originally proposed and widely used to explore the predictability of realized volatility [2,3,4,5]. In particular, Anderson et al. [2] used the HAR models for volatility prediction of stock prices, foreign exchange rates, and bond prices. Corsi et al. [3] discussed the volatility of the realized volatility based on HAR models with non-Gaussianity and volatility clustering. McAleer and Medeiros [4] proposed an extension of the HAR model with a multiple-regime smooth transition which contains long memory and nonlinearity, and incorporates sign and size asymmetries. Hillebrand and Medeiros [5] considered log–linear and neural network HAR models of realized volatility. Tang and Chi [6] found that the HAR model showed better predictive ability than the ARFIMA-RV model. Clements et al. [7], Bollerslev et al. [8], Bianco et al. [9], and Asai et al. [10] investigated successful uses of the HAR models for risk management with VaR measures, risk-return tradeoff, serial correlation, implied volatility, and realized volatility errors. Luo et al. [11] incorporated jumps, leverage effects, and speculation effects into the realized volatility modeling and showed that the portfolio using infinite hidden Markov regime-switching HAR model achieves higher portfolio returns than benchmark HAR model. Meanwhile, as an application of the HAR-RV model to various financial data such as oil, gold, and bitcoin realized volatility [12,13,14,15], we considered extensions of the model incorporating with an associated-uncertainty index to obtain high forecasting gains.

Along with the success of univariate HAR models as above, a multivariate HAR model has been adopted for financial analysis due to its usefulness with multivariate data. Many researchers discussed the superiority of the multivariate HAR model. Busch et al. [16] used a vector HAR model to control possible endogeneity issues. Taylor [17] demonstrated that the multivariate HAR-RV model improved forecast accuracy of the realized volatility in the international stock market. The claim in [17] was also verified by Hwang and Hong [18], who dealt with the multivariate HAR-RV model with heteroskedastic errors. Cech and Barunik [19] showed the generalized HAR model offers better predictability than univariate models in commodity markets. Tang et al. [20,21] showed that the multivariate HAR-RV model is more accurate in out-of-sample forecasting and outperforms the univariate models.

In addition, the multivariate HAR model represents strong correlations between the multiple assets data and examines cross-market spillover effects. For instance, Bubak et al. [22] used a multivariate extension of the HAR model to analyze volatility transmission between currencies and foreign exchange rates, whereas Bauer and Vorkink [23] adopted a multivariate setting of the HAR model and showed how to ensure positive covariance matrix without parameter restrictions. Soucek and Todorova [24] found instantaneous correlation between equity and energy futures by proposing a vector HAR model. Cubadda et al. [25] studied a vector HAR index model for detecting the presence of commonalities in a set of realized volatility measures, whereas Bollerslev et al. [26] proposed a model for a scalar version of vectorized HAR model for the variances and correlations separately. Luo and Ji [27] combined the HAR model with other models to identify time-varying volatility connectedness. Luo and Chen [28] employed matrix log transformation method to ensure the positive definiteness of covariance matrices and developed a Bayesian random compressed multivariate HAR model to forecast the realized covariance matrices of stock returns. Wilms et al. [29] showed that cross-market spillover effects embedded in the multivariate HAR models have long-term forecasting power.

Even though the HAR model is widely used for volatility forecasting based on the realized volatility of intraday prices, it is not restricted to the realized volatility but can be applied to various time series data such as the stock price itself or other economics index, because the HAR model is theoretically a linear AR model. Stock market forecasting techniques were surveyed in [30,31,32], including stock returns, stock prices, and volatility via conventional time series methods and soft computing methods. Stock price modelings are mostly based on efficient market hypothesis (EMH), random walk theory and machine learning techniques as in [33,34,35,36]. According to the EMH, the only relevant information on the stock is its current values. A promising application of the HAR model could be the stock price movement. A reason why the HAR model is expected to perform well on the stock price modeling is that the current value itself and the current averages make its future value in the model.

In this work, we propose a multivariate time series model for strongly correlated data and study its statistical inference of hypothesis test and estimation, with empirical analysis on joint data of financial assets. More specifically, we focus on the multivariate HAR model with exponentially decaying coefficients for the application to stock prices in the stock market. Because two or more financial data exhibit a similar pattern with strong correlation, a multivariate model should be adopted for the multiple assets, instead of univariate models for each asset. However, when the multivariate HAR model is employed to analyze the multiple data, there are many parameters to be estimated. For example, even with two assets, a bivariate HAR(3) model has 14 parameters including two intercept terms. For better performance, we need to make some efforts to reduce errors along with fewer parameters in the model. As a trial for this, we consider the exponentially weighted multivariate HAR model that has exponentially decaying coefficients. If decay rates can be imposed in the multivariate HAR model, the number of parameters substantially decreases and the proposed model might outperform the existing models, reducing the errors as well. This is one of the motivations for this work in the spirit of a principle of parsimony. Moreover, the decay rates not only serve as the long-memory effect as seen in [37], but also represent the commonality of the joint data, as we expect a common structure in multiple assets with strong correlation.

In order to employ the proposed model for joint time series data, the data need to be tested before fitting the model. To this end, we deal with a test problem based on the CUSUM test to identify the presence of decay coefficients in the multivariate HAR model of the fitted data. In general, the CUSUM test is a change-point test and would be reasonable if parameter changes are expected within the time series. For example, Refs. [38,39] dealt with CUSUM(SQ) tests for mean and variance change-detection in univariate HAR(*∞*) models and [40] proposed a CUSUM test for parameter change in dynamic panel models. However, the idea of the CUSUM tests can be applied to detect other dynamic structures. In this work, we suggest the use of such an idea to detect coefficient structure by generating pseudo-time series of residuals in two versions. In other words, by applying the idea of the tests to the difference series of two types of residuals, but not to the original data, the coefficient structure can be identified. That is, the CUSUM(SQ) tests of mean and variance change-detection in [38,39] are used for the pseudo-time series generated by two residuals. The key point is that under the null hypothesis, the mean or variance of the difference series are not changed over time, whereas under the alternative hypothesis there exist change-points in mean or variance of the difference series of two residuals. This idea is a novel attempt in that the CUSUM tests are used for other test problems in time series analysis, not limited to the conventional change-point detection of the raw data.

This work proposes two CUSUM-based tests to detect whether the underlying model has exponentially decaying coefficients. The first test is conducted to test whether the model has an exponential decay rate for each asset, and the second tests whether the exponentially weighted multivariate HAR model has a common decay rate for all the multiple assets. The null limiting distributions are developed as the standard Brownian bridge, and the theoretical results are proven by means of a modified version of a martingale central limit theorem. Additionally, easy-to-implement estimators of the decay rates are discussed.

A Monte Carlo experiment is carried out to see the sample paths of our model and to validate the proposed statistical methods. The sample paths depict the long-memory feature as well as strong cross-correlation of the simulated data. Furthermore, various related series such as difference series and test statistics are depicted to justify our proposed tests under the null and alternative hypotheses. The simulation study not only strongly supports the proposed CUSUM tests with reasonable performances of size and power, but also shows consistency in estimates of decay rates. To compare with the conventional HAR model, root mean squared error (RMSE), mean absolute error (MAE), AIC, and BIC are evaluated in the models with several values of fitting parameters as well as efficiency of the exponentially weighted HAR model, relative to the benchmark HAR model, is computed by using two metrics of RMSE and MAE. It is reported that our proposed model with fewer parameters can reduce the residual errors, compared to the existing HAR models.

As an empirical application of this work, financial market stock prices with similar patterns are selected to suit the multivariate HAR model. It is interesting that the exponentially weighted multivariate HAR model is shown to be suitable for the joint data of U.S. stock prices, rather than the volatility. Our proposed CUSUM tests favor the existence of the decay rates in the multivariate HAR model of the stock prices, based on the computed test statistics. The decay-rate estimators for the stock prices are evaluated as well. The stock prices are well-matched to the exponentially weighted multivariate HAR model. To compare performance of the proposed model, RMSE, MAE, AIC, and BIC are evaluated along with those of the conventional univariate and multivariate HAR models via OLSE and LASSO. The exponentially weighted multivariate HAR model outperforms others in the chosen datasets of U.S. stock prices.

We summarize main benefits of the exponentially weighted multivariate HAR model according to the following points: fewer number of parameters, reduction of the model-fitting errors, representation of the common structure with decay rates, and an appropriate model for joint datasets of stock prices with similar patterns. Our proposed model is suitable for strongly cross-correlated multivariate (bivariate) data with similar patterns because the decay rates yield a common structure in the joint data. Along with the high applicability of the HAR model, the proposed model can be used to analyze and forecast the joint data with strong correlation and long memory as well as its extension can be considered with an exogenous variable such as associated-uncertainty index, as in [12,13,14,15]. The proposed model would help analysts provide simpler and more efficient models by producing smaller errors in predictions in financial time series. Furthermore, it has the potential to extend to dynamic time series models with error terms of heteroscedasticity, time-varying variance, non-Gaussianity, or heavy-tailed distribution, which are more practical in real-world financial markets.

The remainder of the paper is organized as follows. In Section 2 we describe the model and develop main results of the tests, and in Section 3 a simulation study is performed. In Section 4, empirical examples are given. Concluding remarks are stated in Section 5, and proofs are drawn in the Appendix A.

## 2. Model and Main Results

We consider a multivariate HAR(p,q) model $\left\{Y_{j,t}:t\in Z,j=1,2,\cdots ,q\right\}$ of order *p* with *q* multiple assets, given by

(1) $$Y_{j,t}=\beta_{j0}+\sum_{i=1}^{p}\beta_{j1}^{\left(i\right)}Y_{1,t-1}^{\left(i\right)}+\cdots +\sum_{i=1}^{p}\beta_{jq}^{\left(i\right)}Y_{q,t-1}^{\left(i\right)}+\epsilon_{j,t}$$

where $Y_{j,t-1}^{\left(i\right)}=\frac{1}{h_{i}}\left(Y_{j,t-1}+\cdots +Y_{j,t-h_{i}}\right)$ with positive integers $\left\{h_{i},\;i=1,2,\cdots ,p\;\right\}$ satisfying $1=h_{1}<h_{2}<\cdots <h_{p}<\infty$, $\left\{\beta_{j0},\beta_{jk}^{\left(i\right)}:\;j,k\in \left\{1,\cdots ,q\right\};\;i\in \left\{1,2,\cdots ,p\right\}\;\right\}$ are parameters to be estimated, and $\left\{\epsilon_{j,t},\;t\in Z,j=1,\cdots ,q\right\}$ are independent random variables with mean zero and finite variance.

**Figure 1:**
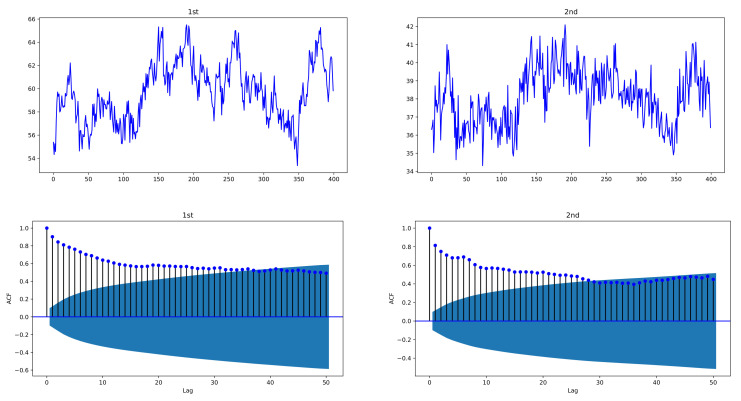
Sample paths of exponentially weighted bivariate HAR model and their ACFs, with decay rates $\lambda_{1}=0.1$, $\lambda_{2}=0.3$; $\left(\beta_{10},c_{11},c_{12}\right)=\left(1.0,0.6,0.45\right)$, $\left(\beta_{20},c_{21},c_{22}\right)=\left(3,0.2,0.35\right)$; n=400. The simulated data of the exponentially weighted bivariate HAR model are characterized with strong cross-correlation and long memory.

**Figure 2:**
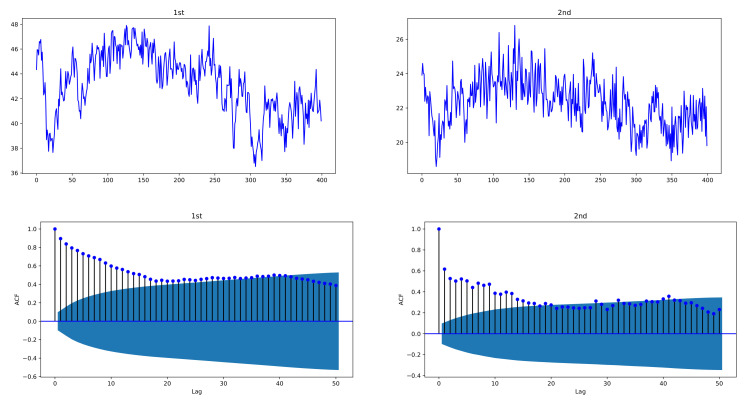
Sample paths of exponentially weighted bivariate HAR model and their ACFs, with common decay rate $\lambda_{1}=\lambda_{2}=0.15$; $\left(\beta_{10},c_{11},c_{12}\right)=\left(1.0,0.6,0.45\right)$, $\left(\beta_{20},c_{21},c_{22}\right)=\left(3,0.2,0.35\right)$; n=400. The simulated data of the exponentially weighted bivariate HAR model are characterized with strong cross-correlation and long memory.

In this work, we are particularly concerned with the multivariate HAR model with exponentially decaying coefficients in order to account for the lesser weights on the farther past values. In the conventional HAR model, regressors are previous value, weekly average and monthly average of consecutive data, which are assigned with coefficients in a decreasing order to represent the long-memory features. For example, see [37], which introduced the (univariate) HAR(∞) model with coefficients decaying exponentially to capture the genuine long-memory. They showed that exponentially decaying coefficients make algebraic decreasing autocovariance functions under appropriate lag conditions in the HAR(*∞*) model. Likewise, we consider the exponentially weighted coefficient version of model (1) with multiple assets, called exponentially weighted multivariate HAR(p,q) model. In our proposed model, coefficients are assumed to be

$$\beta_{jk}^{\left(i\right)}=c_{jk}\lambda_{j}^{i-1}\;\mathrm{for}\;\mathrm{some}\;c_{jk}\;\mathrm{and}\;0<\left|\lambda_{j}\right|<1,$$

for j,k∈{1,⋯,q} and i∈{1,⋯,p}. The $c_{jk}$ is the first coefficient for the previous value of the *k*th asset, $Y_{k,t-1}$, at the first lag t−1, and the $\lambda_{j}$ is the decay rate for the next coefficients. The exponentially weighted multivariate HAR model has long memory as seen in Figure 1 and Figure 2 in the next section, where autocovariance functions as well as the sample paths of the model with decay rates are observed. The decay rates $\lambda_{j}$ not only clearly represent the long-memory feature but also reduce the number of parameters to estimate. In this work, we mainly focus on detecting the existence of the decay rates in model (1) and additionally deal with estimating the decay rates.

In the multivariate HAR model, we first study the hypothesis test problem whether the underlying model is an exponentially weighted multivariate HAR model with decay rates, and secondly we handle easy-to-implement estimators of the decay rates.

For the hypothesis test problem, we consider two tests in (i) and (ii) as follows:

(i) whether or not, in the multivariate HAR model, the *j*th asset has a decay rate $\lambda_{j}$ satisfying $\beta_{jk}^{\left(i\right)}=c_{jk}\lambda_{j}^{i-1}$ for some $c_{jk}$ for each *j*;
(ii) whether or not the exponentially weighted multivariate HAR model has a common rate λ for all multiple assets, i.e., $\beta_{jk}^{\left(i\right)}=c_{jk}\lambda^{i-1}$ for some 0<|λ|<1 for all j,k.

In test (i), each asset is first individually analyzed. Once test (i) has been conducted to favor the null, test (ii) is performed to detect a common rate. For test (i), the null hypothesis $H_{j,0}$ and the alternative hypothesis $H_{j,A}$ are, for each *j*, stated as

$$\left\{\begin{array}{cc} H_{j,0}: & \beta_{jk}^{\left(i\right)}=c_{jk}\lambda_{j}^{i-1}\;\mathrm{for}\;\mathrm{some}\;c_{jk}\;\mathrm{and}\;0<\left|\lambda_{j}\right|<1,\;\;\;\mathrm{vs}.\\ H_{j,A}: & \;\mathrm{the}\;\mathrm{model}\;\mathrm{is}\;\mathrm{not}\;\mathrm{the}\;\mathrm{exponentially}\;\mathrm{weighted}\;\mathrm{HAR}\;\mathrm{model}. \end{array}\right.$$

In order to introduce a test statistic, we adopt the ordinary least squares estimator (OLSE) of the multivariate HAR model. Suppose that we have observed $\left\{Y_{j,t}:-h_{p}+1\leq t\leq n,j=1,2,\cdots ,q\right\}$ of sample size *n*. Let the OLSE of $\beta_{j}\equiv (\beta_{j0},\beta_{j1}^{\left(1\right)},\cdots ,\beta_{j1}^{\left(p\right)},\cdots ,\beta_{jq}^{\left(1\right)},$ $,\cdots ,\beta_{jq}^{\left(p\right)}{)}^{⊤}$ be denoted by

$${\hat{\beta }}_{j}\equiv {\left({\hat{\beta }}_{j0},{\hat{\beta }}_{j1}^{\left(1\right)},\cdots ,{\hat{\beta }}_{j1}^{\left(p\right)},\cdots ,{\hat{\beta }}_{jq}^{\left(1\right)},\cdots ,{\hat{\beta }}_{jq}^{\left(p\right)}\right)}^{⊤}.$$

The asymptotic property of OLSE ${\hat{\beta }}_{j}$ in the multivariate HAR model is derived theoretically by Hong et al. [41]. From the OLSE, we first choose an estimate of $c_{jk}$ by ${\hat{c}}_{jk}={\hat{\beta }}_{jk}^{\left(1\right)}$, and then consider a regression model with the decay rates as its coefficients under the null hypothesis as in (2) and (4). To describe the regression model, we let

$$\eta_{j,t}=Y_{j,t}-\beta_{j0}-\sum_{k=1}^{q}c_{jk}Y_{k,t-1}^{\left(1\right)},\;\;\;\;{\hat{\eta }}_{j,t}=Y_{j,t}-{\hat{\beta }}_{j0}-\sum_{k=1}^{q}{\hat{c}}_{jk}Y_{k,t-1}^{\left(1\right)}.$$

Note that under the null hypothesis,

$$\eta_{j,t}=c_{j1}\left(\sum_{i=2}^{p}\lambda_{j}^{i-1}Y_{1,t-1}^{\left(i\right)}\right)+\cdots +c_{jq}\left(\sum_{i=2}^{p}\lambda_{j}^{i-1}Y_{q,t-1}^{\left(i\right)}\right)+\epsilon_{j,t}.$$

We rewrite $\eta_{j,t}$ as follows:

(2) $$\eta_{j,t}=\lambda_{j}W_{j,t-1}^{\left(2\right)}+\cdots +\lambda_{j}^{p-1}W_{j,t-1}^{\left(p\right)}+\epsilon_{j,t},$$

where $W_{j,t-1}^{\left(i\right)}=c_{j1}Y_{1,t-1}^{\left(i\right)}+\cdots +c_{jq}Y_{q,t-1}^{\left(i\right)}$, for i=2,3,⋯,p. Let

(3) $${\hat{W}}_{j,t-1}^{\left(i\right)}={\hat{c}}_{j1}Y_{1,t-1}^{\left(i\right)}+\cdots +{\hat{c}}_{jq}Y_{q,t-1}^{\left(i\right)},$$

and consider the following regression in (4) with coefficients $\lambda_{j,1},$⋯,$\lambda_{j,p-1}$, which is a similar form to (2) but replaced by observable quantities ${\hat{\eta }}_{j,t}$ and ${\hat{W}}_{j,t-1}^{\left(k\right)}$, k=2,⋯,p:

(4) $${\hat{\eta }}_{j,t}=\lambda_{j,1}{\hat{W}}_{j,t-1}^{\left(2\right)}+\cdots +\lambda_{j,p-1}{\hat{W}}_{j,t-1}^{\left(p\right)}+\epsilon_{j,t}.$$

From this regression we compute OLSE ${\hat{\Lambda }}_{j,n}$ of the parameters $(\lambda_{j,1},\cdots$$,\lambda_{j,p-1}{)}^{⊤}$ by ${\hat{\Lambda }}_{j,n}={\left({\hat{\lambda }}_{j,1},\cdots ,{\hat{\lambda }}_{j,p-1}\right)}^{⊤}.$ Note that under the null hypothesis with $\beta_{jk}^{\left(i\right)}=c_{jk}\lambda_{j}^{i-1}$, it follows that

(5) $$|{\hat{\lambda }}_{j,i-1}-{\hat{\lambda }}_{j,1}^{i-1}|\to^{p}0.$$

Thus, to construct a test statistic, two types of residuals $\left\{{\hat{\epsilon }}_{j,t}\right\}$ and $\left\{{\tilde{\epsilon }}_{j,t}\right\}$ are respectively defined by

$$\begin{array}{ccc} {\hat{\epsilon }}_{j,t} & = & {\hat{\eta }}_{j,t}-\left({\hat{\lambda }}_{j,1}{\hat{W}}_{j,t-1}^{\left(2\right)}+{\hat{\lambda }}_{j,2}{\hat{W}}_{j,t-1}^{\left(3\right)}+\cdots +{\hat{\lambda }}_{j,p-1}{\hat{W}}_{j,t-1}^{\left(p\right)}\right),\\ {\tilde{\epsilon }}_{j,t} & = & {\hat{\eta }}_{j,t}-\left({\hat{\lambda }}_{j,1}{\hat{W}}_{j,t-1}^{\left(2\right)}+{\hat{\lambda }}_{j,1}^{2}{\hat{W}}_{j,t-1}^{\left(3\right)}+\cdots +{\hat{\lambda }}_{j,1}^{p-1}{\hat{W}}_{j,t-1}^{\left(p\right)}\right). \end{array}$$

To construct a test statistic, we use the difference series of the two types of residuals, (not the original time series). Let

(6) $$D_{j,t}\equiv D_{j,t,n}={\tilde{\epsilon }}_{j,t}-{\hat{\epsilon }}_{j,t}=\left({\hat{\lambda }}_{j,2}-{\hat{\lambda }}_{j,1}^{2}\right){\hat{W}}_{j,t-1}^{\left(3\right)}+\cdots +\left({\hat{\lambda }}_{j,p-1}-{\hat{\lambda }}_{j,1}^{p-1}\right){\hat{W}}_{j,t-1}^{\left(p\right)}.$$

Let $S_{j,n}=\frac{1}{{\hat{\sigma }}_{j,D}\sqrt{n}}\sum_{t=1}^{n}D_{j,t}$ where ${\hat{\sigma }}_{j,D}^{2}$ is a consistent estimator of $Var(D_{j,t})$, for example,

(7) $${\hat{\sigma }}_{j,D}^{2}=\frac{1}{n}\sum_{t=1}^{n}D_{j,t}^{2}\;\;\;\;\mathrm{or}\;\;\;{\hat{\sigma }}_{j,D}^{2}=\frac{1}{n}\sum_{t=1}^{n}D_{j,t}^{2}-{\left(\frac{1}{n}\sum_{t=1}^{n}D_{j,t}\right)}^{2},$$

noting that $E[D_{j,t}]\to 0$ as n→∞ under the null hypothesis. Now we define a CUSUM test statistic ${\hat{T}}_{j,n}\left(z\right)$ as follows: for 0≤z≤1,

(8) $${\hat{T}}_{j,n}\left(z\right)=\frac{1}{{\hat{\sigma }}_{j,D}\sqrt{n}}\left[\sum_{t=1}^{\left[nz\right]}D_{j,t}-z\sum_{t=1}^{n}D_{j,t}\right].$$

The following theorem states asymptotic distribution of both statistics. It provides critical values of the test for $H_{j,0}$.

**Theorem** **1.** *We assume $E|Y_{j,t}{|}^{2+\delta }<\infty$ for some δ>0, for all j,t. If the multivariate HAR(p.q) model has exponential decay rate $\lambda_{j}$ with $\beta_{jk}^{\left(i\right)}=c_{jk}\lambda_{j}^{i-1}$ for some $0<|\lambda_{j}|<1$ for each j, then we have, as n→∞,*

$$S_{j,n}\to^{d}N\left(0,1\right)\;\;\;\;and\;\;\;\;\underset{0\leq z\leq 1}{\sup}|{\hat{T}}_{j,n}\left(z\right)|\to^{d}\underset{0\leq z\leq 1}{\sup}\left|B_{0}\left(z\right)\right|$$

*where $B_{0}\left(z\right)=B\left(z\right)-zB\left(1\right)$ is the standard Brownian bridge with the Brownian motion B(z).*

**Figure 3:**
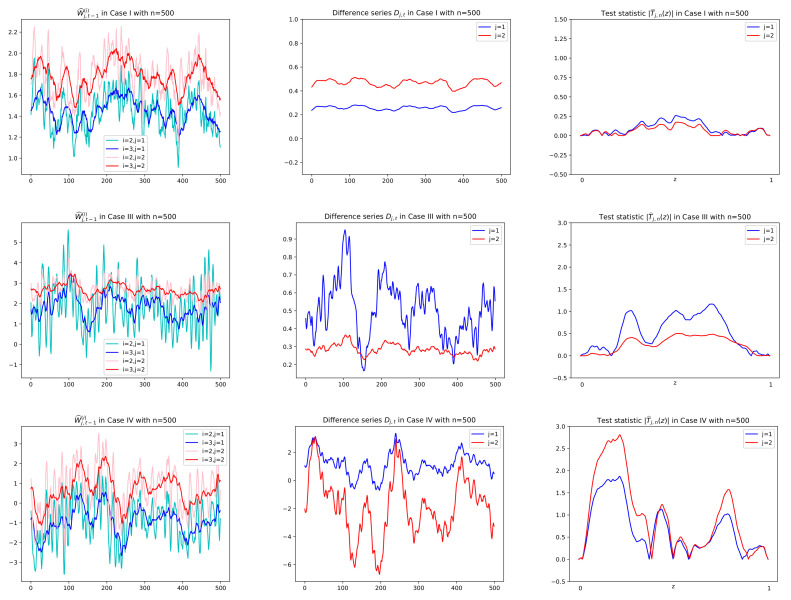
Sample paths of ${\hat{W}}_{j,t-1}^{\left(i\right)},D_{j,t}$ and $|{\hat{T}}_{j,n}\left(z\right)|$, (j=1,2;i=2,3) in Cases I, III, IV in Theorem 1 with n=500. Difference series $D_{j,t}$ in the second column are obtained by multiplying $W_{j,t-1}^{\left(3\right)}$ in the first column by ${\hat{\lambda }}_{j,2}-{\hat{\lambda }}_{j,1}^{2}$ given in Figure 5. Test statistics in absolute $|{\hat{T}}_{n,j}\left(z\right)|$, (0≤z≤1), are computed using the difference series $D_{j,t}$ in the second column. In the first row of Case I with both $H_{1,0}$ and $H_{2,0}$, $D_{j,t}$ has no change in mean and thus small values of $|{\hat{T}}_{n,j}\left(z\right)|$ for all *z*. In the second row of Case III with $H_{2,0}$, j=2 (in red), the same interpretation is given.

**Figure 4:**
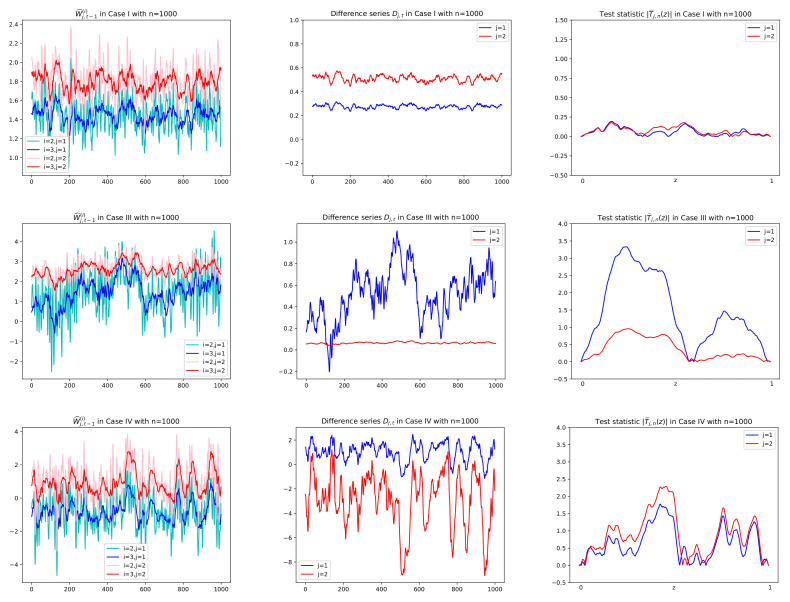
Sample paths of ${\hat{W}}_{j,t-1}^{\left(i\right)},D_{j,t}$ and $|{\hat{T}}_{j,n}\left(z\right)|$, (j=1,2;i=2,3) in Cases I, III, IV in Theorem 1 with n=1000. Difference series $D_{j,t}$ in the second column are obtained by multiplying $W_{j,t-1}^{\left(3\right)}$ in the first column by ${\hat{\lambda }}_{j,2}-{\hat{\lambda }}_{j,1}^{2}$ given in Figure 5. Test statistics in absolute $|{\hat{T}}_{n,j}\left(z\right)|$, (0≤z≤1), are computed using the difference series $D_{j,t}$ in the second column. In the first row of Case I with both null hypotheses $H_{1,0}$ and $H_{2,0}$, $D_{j,t}$ has no change in mean and thus small values of $|{\hat{T}}_{n,j}\left(z\right)|$ for all *z*. In the second row of Case III with $H_{2,0}$, j=2 (in red), the same interpretation is given.

**Remark** **1.** *In order to test $H_{j,0}$ vs. $H_{j,A}$, we adopt the CUSUM test statistics ${\hat{T}}_{j,n}\left(z\right)$, rather than $S_{j,n}$, and the null hypothesis is rejected if $|{\hat{T}}_{j,n}\left(z\right)|$ is large. The reason is as follows: The difference series $\left\{D_{j,t}\right\}$ has coefficients $\left\{{\hat{\lambda }}_{j,i}-{\hat{\lambda }}_{j,1}^{i}:i=2,3,\cdots ,p-1\right\}$ in the linear combination of $\left\{{\hat{W}}_{j,t-1}^{\left(i\right)}:i=3,\cdots ,p\right\}$. Note that the pseudo-time series $\left\{D_{j,t}=D_{j,t,n}:t=1,2,\cdots ,n\right\}$ is a triangular array and under the null hypothesis the coefficients are asymptotically zeros whereas under the alternative hypothesis the coefficients are changed over the time without vanishing asymptotically, which makes a change-point in mean or variance of the difference series. This idea is the reason why we adopt the CUSUM-based test for our goal that is to detect the exponentially decay rates. Sample paths of the series ${\hat{W}}_{j,t-1}^{\left(i\right)}$ and $D_{j,t}$ under both $H_{j,0}$ and $H_{j,A}$ can be seen in Figure 3 and Figure 4 along with values of $|{\hat{T}}_{j,n}\left(z\right)|$. In Figure 3 and Figure 4, it is shown that the difference series under the null is an asymptotical constant due to the asymptotical zeros of the coefficients, whereas under the alternative, it fluctuates with large variance; that is, it indicates that there are change-points in mean or variance. On the other hand, we might use the full sum $S_{j,n}$ as a test statistic in a view of theoretical insight. However, as seen in Figure 3 and Figure 4, even under $H_{j,0}$, the sum is not evaluated as small values because of the following reason: $S_{j,n}$ can be expressed as a linear combination of $\left\{\sum_{t=1}^{n}{\hat{W}}_{j,t-1}^{\left(i\right)}/\sqrt{n}:i=3,\cdots ,p\right\}$ and thus as a linear combination of $\left\{\sum_{t=1}^{n}{\hat{c}}_{jk}Y_{k,t-1}^{\left(i\right)}/\sqrt{n}:i=3,\cdots ,p;\;k=1,\cdots ,q\right\}$. Note that for each k, $\sum_{t=1}^{n}\left(Y_{k,t-1}^{\left(i\right)}-E\left[Y_{k,t-1}^{\left(i\right)}\right]\right)/\sqrt{n}$ converges to the normal distribution with asymptotic mean zero. Thus $S_{j,n}$ makes an asymptotic bias of the form $\sqrt{n}\left({\hat{\lambda }}_{j,k}-{\hat{\lambda }}_{j,1}^{k}\right)E\left[Y_{k,t-1}^{\left(i\right)}\right]$ in a finite sample. Because the asymptotic bias is not negligible even though $\sqrt{n}\left({\hat{\lambda }}_{j,k}-{\hat{\lambda }}_{j,1}^{k}\right)$ tends to normal distribution with mean zero under the null hypothesis, the sum $S_{j,n}$ has somewhat large values and thus cannot distinguish significantly the two hypotheses. Therefore, this work adopts the test statistic ${\hat{T}}_{j,n}\left(z\right)$ to resolve our problem.*

Now we would further like to test whether or not the exponential weighted multivariate HAR model has a common exponential decay rate λ for all multiple assets. That is, in the exponentially weighted multivariate HAR(p,q) model with $\beta_{jk}^{\left(i\right)}=c_{jk}\lambda_{j}^{i-1}$ for some $0<|\lambda_{j}|<1$ for all j,k, after the first test has been performed, we test the null hypothesis $H_{0}^{*}$ versus the alternative hypothesis $H_{A}^{*}$ as follows:

$$\left\{\begin{array}{cc} H_{0}^{*}: & \lambda_{1}=\cdots =\lambda_{q}=\lambda \;\mathrm{with}\;a\;\mathrm{common}\;\mathrm{rate}\;\lambda \;\mathrm{on}\;\mathrm{all}\;\mathrm{assets}\;j,\\ H_{A}^{*}: & \;\mathrm{not}\;\mathrm{the}\;\mathrm{null}. \end{array}\right.$$

Similar to the above, under the null $H_{0}^{*}$, for all *j* we have $\eta_{j,t}=\lambda W_{j,t-1}^{\left(2\right)}+\cdots +\lambda^{p-1}W_{j,t-1}^{\left(p\right)}+\epsilon_{j,t}$ instead of (2), but we use a consistent estimate ${\hat{\lambda }}_{j}$ of $\lambda_{j}$ for $\left\{Y_{j,t}\right\}$. For the estimation of the rates $\lambda_{j}$, j=1,⋯,q, we discuss below in Remark 2. By using the estimate ${\hat{\lambda }}_{j}$ we compute residuals ${\hat{\epsilon }}_{j,t}^{*}={\hat{\eta }}_{j,t}-\left({\hat{\lambda }}_{j}{\hat{W}}_{j,t-1}^{\left(2\right)}+\cdots +{\hat{\lambda }}_{j}^{p-1}{\hat{W}}_{j,t-1}^{\left(p\right)}\right)$ for each *j*. Now, we let

$$\epsilon_{t}^{*}\left(j\right)=\underset{k\neq j}{\sup}\left[{\hat{\eta }}_{j,t}-\left({\hat{\lambda }}_{k}{\hat{W}}_{j,t-1}^{\left(2\right)}+\cdots +{\hat{\lambda }}_{k}^{p-1}{\hat{W}}_{j,t-1}^{\left(p\right)}\right)\right]$$

and let $d_{j,t}={\hat{\epsilon }}_{j,t}^{*\;2}-\epsilon_{t}^{*}{\left(j\right)}^{2}$, ${\tilde{D}}_{j,t}=d_{j,t}^{2}-{\hat{\sigma }}_{j,d}^{2}$ where ${\hat{\sigma }}_{j,d}^{2}=\sum_{t=1}^{n}d_{j,t}^{2}/n$. Also, let $D_{t}^{*}=\sum_{j=1}^{q}{\tilde{D}}_{j,t}$. We construct a test statistic for testing if the HAR model has common rate as follows:

For 0≤z≤1, let ${\hat{T}}_{n}^{*}\left(z\right)=\sum_{t=1}^{\left[nz\right]}D_{t}^{*}/\left({\hat{\sigma }}_{D}^{*}\sqrt{n}\right),$ which is rewritten as

$${\hat{T}}_{n}^{*}\left(z\right)=\frac{1}{{\hat{\sigma }}_{D}^{*}\sqrt{n}}\sum_{j=1}^{q}\left[\sum_{t=1}^{\left[nz\right]}d_{j,t}^{2}-z\sum_{t=1}^{n}d_{j,t}^{2}\right]$$

where ${\hat{\sigma }}_{D}^{*2}$ is a consistent estimator of $Var(D_{t}^{*})$ such as $\frac{1}{n}\sum_{t=1}^{n}D_{t}^{*2}.$ The following theorem provides the null limiting distribution of the test statistic.

**Theorem** **2.** *We assume $E|Y_{j,t}{|}^{2+\delta }<\infty$ for some δ>0, for all j,t. If the multivariate HAR(p.q) model has a common exponential decay rate λ with $\beta_{jk}^{\left(i\right)}=c_{jk}\lambda^{i-1}$ for some 0<|λ|<1 for all j,k, then we have, as n→∞,*

$$\underset{0\leq z\leq 1}{\sup}|{\hat{T}}_{n}^{*}\left(z\right)|\to^{d}\underset{0\leq z\leq 1}{\sup}\left|B_{0}\left(z\right)\right|.$$

Note that under the null hypothesis $H_{0}^{*}$, the difference series $\left\{d_{j,t}\right\}$ are evaluated as small values and are characterized with small variance, but under the alternative hypothesis $H_{A}^{*}$, they have large values with dynamic variance over the time. Thus we use the CUSUMSQ test for the difference series $\left\{d_{j,t}\right\}$ (not the original data) to see the change-point of the variance. Justification of suitability of the CUSUMSQ test can be seen in the next section, where sample paths of the difference squared, $d_{j,t}^{2}$, and the values of test statistics in absolute, $|{\hat{T}}_{n}^{*}\left(z\right)|$, under both hypotheses are depicted.

Once the first test in Theorem 1 has been conducted to datasets of multiple assets, we obtain estimates of the decay rates by using (9), and then the second test in Theorem 2 is conducted to see whether the datasets have a common rate. Finally, the estimate (10) is used to find the common rate.

**Remark** **2.** *The following concerns the estimation of the decay rates. In the exponentially weighted multivariate HAR model (1) with coefficients $\beta_{jk}^{\left(i\right)}=c_{jk}\lambda_{j}^{i-1}$, estimators of the decay rates $\lambda_{j}$ can be obtained in a simple way. From the OLSEs of parameter vector $\beta_{j}$, we construct an easy-to-implement estimator of $\lambda_{j}$ as follows:*

(9) $${\hat{\lambda }}_{j}=\frac{\sum_{k=1}^{q}{\hat{\beta }}_{jk}^{\left(2\right)}}{\sum_{k=1}^{q}{\hat{\beta }}_{jk}^{\left(1\right)}}.$$

*Furthermore, in case of the common rate with $\beta_{jk}^{\left(i\right)}=c_{jk}\lambda^{i-1}$, the common rate λ is estimated by*

(10) $$\hat{\lambda }=\frac{\sum_{j=1}^{q}\sum_{k=1}^{q}{\hat{\beta }}_{jk}^{\left(2\right)}}{\sum_{j=1}^{q}\sum_{k=1}^{q}{\hat{\beta }}_{jk}^{\left(1\right)}}.$$

*In the estimates of the decay rates in (9) and (10), only the first and the second coefficients estimates, i.e., ${\hat{\beta }}_{jk}^{\left(1\right)}$ and ${\hat{\beta }}_{jk}^{\left(2\right)}$, are used. This is because these two estimates have comparatively fewer standard errors than others. To see their performances, sample means and standard errors of the estimates in (9) and (10) are computed and compared in the next section.*

In the conventional multivariate HAR(p,q) model there are a total of (1+pq)q coefficient parameters to estimate, whereas in the exponential weighted multivariate HAR(p,q) model the number of parameters is decreased to (2+q)q. Each j=1,2,⋯,q, $Y_{j,t}$ has one intercept, and *q* coefficients of the previous lag values of *q* assets and one decay rate. For a simple case with p=3 and q=2, the number of parameters is reduced from 14 to 8. This implies that some measures of statistical models such as AIC and BIC might be improved considerably. This improvement can be shown in the following sections with simulated data and real data examples. In the multivariate HAR(p,q) model, the asymptotic normality for the OLSE ${\hat{\beta }}_{j,O}\left(\equiv {\hat{\beta }}_{j,OLSE}\right)$ of $\beta_{j}$ has been established by Hong et al. [41]: $\sqrt{n}\left({\hat{\beta }}_{j,O}-\beta_{j}\right)\to^{d}N\left(0,\Sigma \right)$ as n→∞, where Σ is some (1+pq)×(1+pq) covariance matrix.

**Remark** **3.** *The following concerns the bias adjustment for a finite sample. In our exponential weighted multivariate HAR model with $\beta_{jk}^{\left(i\right)}=c_{jk}\lambda_{j}^{i-1}$, which are components of $\beta_{j}={\left(\beta_{j0},\beta_{j1}^{\left(1\right)},\cdots ,\beta_{j1}^{\left(p\right)},\cdots ,\beta_{jq}^{\left(1\right)},\cdots ,\beta_{jq}^{\left(p\right)}\right)}^{⊤}$, we construct an estimator ${\tilde{\beta }}_{j,\Lambda }$ of $\beta_{j}$, called the rate-adopted estimator (RE), as follows: ${\tilde{\beta }}_{j,\Lambda }={\left({\tilde{\beta }}_{j0},{\tilde{\beta }}_{j1}^{\left(1\right)},\cdots ,{\tilde{\beta }}_{j1}^{\left(p\right)},\cdots ,{\tilde{\beta }}_{jq}^{\left(1\right)},\cdots ,{\tilde{\beta }}_{jq}^{\left(p\right)}\right)}^{⊤}$ where*

$${\tilde{\beta }}_{j0}={\hat{\beta }}_{j0},\;\;{\tilde{\beta }}_{jk}^{\left(1\right)}={\hat{c}}_{jk}\left(\equiv {\hat{\beta }}_{jk}^{\left(1\right)}\right)\;\;and\;\;{\tilde{\beta }}_{jk}^{\left(i\right)}={\hat{c}}_{jk}{\hat{\lambda }}_{j}^{i-1}\;\;for\;\;i\geq 2$$

*with ${\hat{\lambda }}_{j}$ in (9). It is obvious that $\left|{\hat{\beta }}_{jk}^{\left(i\right)}-{\tilde{\beta }}_{jk}^{\left(i\right)}\right|=\left|{\hat{\beta }}_{jk}^{\left(i\right)}-{\hat{c}}_{jk}{\hat{\lambda }}_{j}^{i-1}\right|\to^{p}0$ as n→∞. Here we need to observe the residuals on behalf of the empirical analysis for a finite sample. We rewrite model (1) as $Y_{j,t}=\beta_{j}^{⊤}X_{t-1}+\epsilon_{j,t}$, where*

$$X_{t-1}={\left(1,Y_{1,t-1}^{\left(1\right)},\cdots ,Y_{1,t-1}^{\left(p\right)},Y_{2,t-1}^{\left(1\right)},\cdots ,Y_{2,t-1}^{\left(p\right)},\cdots ,Y_{q,t-1}^{\left(1\right)},\cdots ,Y_{q,t-1}^{\left(p\right)}\right)}^{⊤}\in R^{\left(1+pq\right)}.$$

*Let ${\hat{\epsilon }}_{j,t,O}$ and ${\tilde{\epsilon }}_{j,t,\Lambda }$ be residuals by the OLSE and the RE, respectively:*

$${\hat{\epsilon }}_{j,t,O}=Y_{j,t}-{\hat{\beta }}_{j,O}^{⊤}X_{t-1}\;\;and\;\;{\tilde{\epsilon }}_{j,t,\Lambda }=Y_{j,t}-{\tilde{\beta }}_{j,\Lambda }^{⊤}X_{t-1}.$$

*Note that*

$${\tilde{\epsilon }}_{j,t,\Lambda }=Y_{j,t}-{\hat{\beta }}_{j,O}^{⊤}X_{t-1}+{\left({\hat{\beta }}_{j,O}-{\tilde{\beta }}_{j,\Lambda }\right)}^{⊤}X_{t-1}={\left(\beta_{j}-{\hat{\beta }}_{j,O}\right)}^{⊤}X_{t-1}+{\left({\hat{\beta }}_{j,O}-{\tilde{\beta }}_{j,\Lambda }\right)}^{⊤}X_{t-1}+\epsilon_{j,t}.$$

*Let ${\bar{\mu }}_{j,\Lambda }=\frac{1}{n}\sum_{t=1}^{n}{\tilde{\epsilon }}_{j,t,\Lambda }$, and then by the asymptotic normality of $\sqrt{n}\left(\beta_{j}-{\hat{\beta }}_{j,O}\right)$ with asymptotic mean zero and by noticing that $({\hat{\beta }}_{j,O}-{\tilde{\beta }}_{j,\Lambda })=$*

$${\left(0;0,{\hat{\beta }}_{j1}^{\left(2\right)}-{\hat{c}}_{j1}{\hat{\lambda }}_{j},\cdots ,{\hat{\beta }}_{j1}^{\left(p\right)}-{\hat{c}}_{j1}{\hat{\lambda }}_{j}^{p-1};0,{\hat{\beta }}_{j2}^{\left(2\right)}-{\hat{c}}_{j2}{\hat{\lambda }}_{j}\cdots ;0,{\hat{\beta }}_{jq}^{\left(2\right)}-{\hat{c}}_{jq}{\hat{\lambda }}_{j},\cdots ,{\hat{\beta }}_{jq}^{\left(p\right)}-{\hat{c}}_{jq}{\hat{\lambda }}_{j}^{p-1}\right)}^{⊤},$$

*we have*

$${\bar{\mu }}_{j,\Lambda }=\frac{1}{n}\sum_{t=1}^{n}\left[{\left({\hat{\beta }}_{j,O}-{\tilde{\beta }}_{j,\Lambda }\right)}^{⊤}X_{t-1}\right]+o_{p}\left(1\right)=\frac{1}{n}\sum_{t=1}^{n}\left[\sum_{k=1}^{q}\sum_{i=2}^{p}\left({\hat{\beta }}_{jk}^{\left(i\right)}-{\hat{c}}_{jk}{\hat{\lambda }}_{j}^{i-1}\right)Y_{k,t-1}^{\left(i\right)}\right]+o_{p}\left(1\right).$$

*Even though $|{\hat{\beta }}_{jk}^{\left(i\right)}-{\hat{c}}_{jk}{\hat{\lambda }}_{j}^{i-1}|\to^{p}0$ under the null hypothesis, $\sum_{k=1}^{q}\sum_{i=2}^{p}\left({\hat{\beta }}_{jk}^{\left(i\right)}-{\hat{c}}_{jk}{\hat{\lambda }}_{j}^{i-1}\right)Y_{k,t-1}^{\left(i\right)}$ is not negligible in a small finite sample. Thus we need the bias adjustment for a fitting model in a finite sample. When we fit the exponentially weighted HAR model to real datasets, especially ones with small sample size, the error performances can be improved by means of the bias adjustment. For instance, one way is that the fitted model is shifted by the residual mean ${\bar{\mu }}_{j,\Lambda }$, which is a constant. An alternative way is that the model is shifted by a moving average of residuals, which is a time-varying process, as we define in the following. For a positive integer m and t=1,2,⋯,n, let*

(11) $${\bar{\omega }}_{m,t}=\frac{1}{\tau_{2}-\tau_{1}+1}\sum_{s=\tau_{1}}^{\tau_{2}}{\tilde{\epsilon }}_{j,s,\Lambda }$$

*where $\tau_{1}\equiv \tau_{1}\left(t,m\right)=\max\left\{1,t-m\right\}$ and $\tau_{2}\equiv \tau_{2}\left(t,m\right)=\min\left\{n,t+m\right\}$, (j is omitted in ${\bar{\omega }}_{m,t}$ for notational simplicity). The time-varying process $\left\{{\bar{\omega }}_{m,t},t=1,2,\cdots n\right\}$ determines the error performances of the fitting model shifted by $\left\{{\bar{\omega }}_{m,t}\right\}$. The fitted model with exponential decay rates is now determined by*

(12) $$Y_{j,t}={\hat{F}}_{j,\Lambda }\left(X_{t-1}\right)+\varepsilon_{j,t}$$

*where ${\hat{F}}_{j,\Lambda }\left(X_{t-1}\right)={\tilde{\beta }}_{j,\Lambda }^{⊤}X_{t-1}+{\bar{\omega }}_{m,t}$ and $\varepsilon_{j,t}={\tilde{\epsilon }}_{j,t,\Lambda }-{\bar{\omega }}_{m,t}$. Note that $\frac{1}{n}\sum_{t=1}^{n}\varepsilon_{j,t}=o_{p}\left(1\right)$. Effects of m, called the fitting parameter, on the error performances of (12) will be discussed in the next section.*

## 3. Monte Carlo Simulation

In this section, we first see the plots of sample paths of the proposed model and their autocorrelation coefficient functions (ACFs). Secondly, finite sample validity of the proposed tests is investigated along with the plots of various related series for the justification of the tests. Thirdly, the estimates of the decay rates are computed, and finally comparisons with conventional HAR models are addressed in terms of measures such as RMSE, MAE, AIC, and BIC. Moreover, efficiency of the proposed model vs. the benchmark HAR model is discussed.

In the simulation experiment, to see the plots of the proposed model, simulated data are generated by bivariate exponentially weighted HAR models of order p=3, HAR(3,2) models, with lag structure $h=\left(h_{1},\cdots ,h_{p}\right)=\left(1,5,22\right)$, by using i.i.d. standard normal distributed N(0,1)-errors $\left\{\epsilon_{j,t}\right\}$ and size n=400. In order to avoid the effect of selected initial value in the models, data of size 600 are generated and the first 200 data are deleted to obtain n=400. Figure 1 and Figure 2 depict sample paths with parameters $\left(\beta_{10},c_{11},c_{12}\right)=\left(1.0,0.6,0.45\right)$, $\left(\beta_{20},c_{21},c_{22}\right)=\left(3,0.2,0.35\right)$, together with their ACFs; Figure 1 uses individual decay rates $\lambda_{1}=0.1$, $\lambda_{2}=0.3$ whereas Figure 2 uses common rate λ=0.15. We see that the simulated data are strongly correlated with each other and reveal the long-memory feature. In Figure 1, two datasets have a correlation coefficient of 0.7856, and in Figure 2, the correlation coefficient is 0.6748.

To verify Theorems 1 and 2, we compute the test statistics in the HAR(3,2) model and report their rejection rates in Table 1 and Table 2, respectively. To see the validation of Theorem 1, four combinations of two datasets are assumed as follows:

• *Case I*: Both are exponentially weighted models with $\lambda_{1}=0.5$ and $\lambda_{2}=0.4$.
• *Case II*: The first data set follows an exponentially weighted model with $\lambda_{1}=0.5$ whereas the second is not.
• *Case III*: The second is an exponentially weighted model with $\lambda_{2}=0.4$ whereas the first is not.
• *Case IV*: None of them are exponentially weighted models.

For the null hypothesis of Cases I, II, and III, $\left(\beta_{10},c_{11},c_{12}\right)=\left(1,0.1,0.25\right),\left(\beta_{20},c_{21},c_{22}\right)=\left(3,0.2,0.15\right),\lambda_{1}=0.5,\lambda_{2}=0.4$ are used. For Case IV, there are total 14 (irregular) parameters, and thus their presentation with so many parameters, including those under the alternative hypothesis in Cases II and III, are omitted here, but are available upon request.

**Figure 5:**
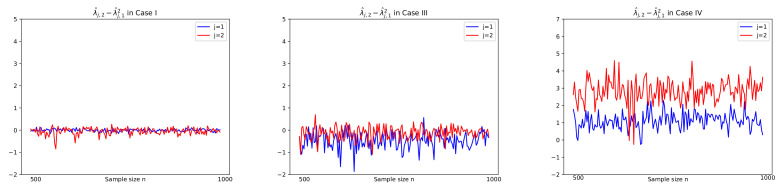
${\hat{\lambda }}_{j,2}-{\hat{\lambda }}_{j,1}^{2}$, (j=1,2), with sample size n=500,501,⋯,1000 on horizontal axis, in Case I, III, IV in Theorem 1. In Case I(j=1,2) and Case III(j=2) with the null hypothesis $H_{j,0}$, ${\hat{\lambda }}_{j,2}-{\hat{\lambda }}_{j,1}^{2}$ tends to zero as n→1000.

Prior to reporting the test results, some plots of related series are illustrated in order to justify the suitability of the CUSUM tests. In particular, for Cases I, III, and IV, sample paths of ${\hat{W}}_{j,t-1}^{\left(i\right)}$, $D_{j,t}$ and $|{\hat{T}}_{j,n}\left(z\right)|$ in (3), (6), and (8), respectively, are depicted with n=500,1000 in Figure 3 and Figure 4, where values of ${\hat{\lambda }}_{j,2}-{\hat{\lambda }}_{j,1}^{2}$ in Figure 5 are used to compute $D_{j,t}$ together with using ${\hat{W}}_{j,t-1}^{\left(i\right)}$. In Figure 5, we see that ${\hat{\lambda }}_{j,2}-{\hat{\lambda }}_{j,1}^{2}$ tends to zero in Case I (j=1,2) and Case III (j=2) as its theory indicates as in (5) under the null hypothesis. However, it is shown in Figure 5 that Case III (j=1) and Case IV (j=1,2) have the deviation from zeros under the alternative. In Figure 3 and Figure 4, under the null hypothesis with decay rates, the difference series $D_{j,t}$ does not fluctuate due to the asymptotic zero of ${\hat{\lambda }}_{j,2}-{\hat{\lambda }}_{j,1}^{2}$, which can be interpreted as constant coefficients in the linear combination of ${\hat{W}}_{j,t-1}^{\left(i\right)}$, whereas under the alternative, plots of $D_{j,t}$ are dynamic with large variance because of nonzero ${\hat{\lambda }}_{j,2}-{\hat{\lambda }}_{j,1}^{2}$, (see Equation (6)). This fact yields higher values of the CUSUM test statistic in absolute, $|{\hat{T}}_{j,n}\left(z\right)|$, as seen in the third columns of Figure 3 and Figure 4.

**Figure 6:**
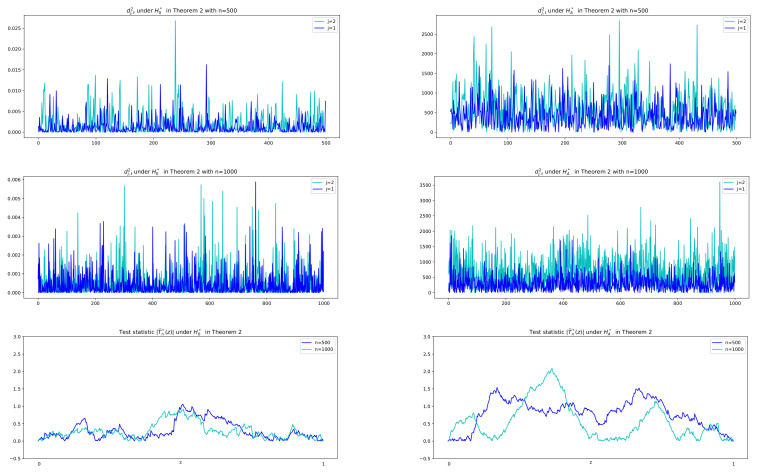
Sample paths of $d_{j,t}^{2}$, (j=1,2), and ${\hat{T}}_{n}^{*}\left(z\right)$ under $H_{0}^{*}$ and $H_{A}^{*}$ in Theorem 2 with n=500,1000. Under $H_{0}^{*}$ in the first column, difference squared $d_{j,t}^{2}$ are small values less than 0.025 for n=500 and 0.006 for n=1000 and thus the test statistic $|{\hat{T}}_{n}\left(z\right)|$ are evaluated as small values less than 1 while under $H_{A}^{*}$ in the second column, $d_{j,t}^{2}$ are between 0 and 2500 and so there exists a variance change-point with large values of $|{\hat{T}}_{n}\left(z\right)|$; $\sup|{\hat{T}}_{n}\left(z\right)|\geq 1.5$.

As for the CUSUMSQ test in Theorem 2, Figure 6 describes series of difference squared, $d_{j,t}^{2}$, and test statistic values in absolute, $|{\hat{T}}_{n}^{*}\left(z\right)|$, under the two hypotheses with n=500,1000, respectively. It is shown that under $H_{0}^{*}$, the difference series $d_{j,t}$ has small variance with small values whose squares are less than 0.025 for n=500 and 0.006 for n=1000, whereas under $H_{A}^{*}$ it has large values with their squares between 0 and 2500. Therefore we adopt the idea of change-point test of variance to the difference series $d_{j,t}$, which yields a solution of the existence of the exponential decay rate.

Throughout the simulation study of the CUSUM tests, replication number 1000, significant level α=0.05, and sample size n=100,200,500, and 1000 are used.

Table 1 and Table 2 display the evaluated rejection rates in Theorems 1 and 2, respectively. In Table 1, results of rejection rates of two tests $H_{j,0}$, j=1,2, in Theorem 1 for the four cases are addressed. To compute the test statistic ${\hat{T}}_{j,n}\left(z\right)$ in Theorem 1, a consistent estimate ${\hat{\sigma }}_{j,D}$ of the standard deviation should be found. Recall two of ${\hat{\sigma }}_{j,D}^{2}$ in (7), which are estimators of $Var\left(D_{j,t}\right)=E\left[D_{j,t}^{2}\right]-E{\left[D_{j,t}\right]}^{2}$. Because $E[D_{j,t}]\to 0$ as n→∞ due to ${\hat{\lambda }}_{j,k}-{\hat{\lambda }}_{j,1}^{k}\to^{p}0$ under the null hypothesis $H_{j,0}$, we may use both in (7) for ${\hat{\sigma }}_{j,D}^{2}$. However, the use of the estimates incurs slow convergence rates because of the bias problem, as seen in the argument in Remark 1. If the mean has a large bias, the convergence rate tends to be slow, as affected by the bias. Thus we adopt the two estimates partially to adjust the convergence rate. In particular, so as to visualize the convergence to the nominal level with increasing *n*, we here take partially the second sample variance in (7) of the form: $\frac{1}{n}\sum_{t=1}^{n}D_{j,t}^{2}-{\left({\bar{D}}_{j}\right)}^{2}$, where ${\bar{D}}_{j}=\frac{1}{n}\sum_{t=1}^{n}D_{j,t}$, which converges to zero in probability along with $|\frac{1}{n}\sum_{t=1}^{n}D_{j,t}-E\left[D_{j,t}\right]|\to^{p}0$ as n→∞. To use it partially, we construct a consistent estimator with the following threshold: ${\tilde{\sigma }}_{j,D}^{2}=\frac{1}{n}\sum_{t=1}^{n}D_{j,t}^{2}-\delta {\left({\bar{D}}_{j}\right)}^{2}$, where $\delta =I(|{\bar{D}}_{j}|<th_{*})$ with an indicator function I(·) and a threshold $th_{*}$. In other words, we use either the first one or the second one in (7), depending on the magnitude of the mean ${\bar{D}}_{j}$. By doing this, we can adjust the convergence rate by adopting the following threshold: setting the value δ of the indicator function to zero in case of the large bias. In this simulation, threshold $th_{*}$ is chosen empirically such that $P(|{\bar{D}}_{j}|<th_{*})=0.05$; that is, if $|{\bar{D}}_{j}|\geq th_{*}$ with probability 0.95, then the first in (7) is used and otherwise with probability 0.05, the second in (7) is used for the estimate ${\tilde{\sigma }}_{j,D}^{2}$. This is because the probability of having the bias is high as seen in Remark 1. In Table 1, results of rejection rates are given by using the estimate ${\tilde{\sigma }}_{j,D}$ with the chosen threshold $th_{*}$ and are seen with the convergence to the nominal level α=0.05 as *n* increases under the null hypothesis. For Table 2 with Theorem 2, a similar argument can be given.

**Table 1:** Validation of Theorem 1. Rejection rate for hypotheses $H_{j,0}$ and $H_{j,A}$ of level α=0.05, j=1,2 in exponentially weighted bivariate HAR models of order p=3; **h** = (1,5,22); $\left(\beta_{10},c_{11},c_{12}\right)=\left(1,0.1,0.25\right),\left(\beta_{20},c_{21},c_{22}\right)=\left(3,0.2,0.15\right),\lambda_{1}=0.5,\lambda_{2}=0.4$ in the null hypothesis $H_{j,0}$ of Cases I, II, III; replication number =1000. (Other parameter values used are available upon request ).

*n*	*Case I*		*Case II*		*Case III*		*Case IV*	
	$H_{1,0}$	$H_{2,0}$	$H_{1,0}$	$H_{2,A}$	$H_{1,A}$	$H_{2,0}$	$H_{1,A}$	$H_{2,A}$
100	0.066	0.056	0.083	0.198	0.547	0.058	0.640	0.776
200	0.053	0.058	0.057	0.236	0.767	0.054	0.789	0.897
500	0.051	0.050	0.057	0.313	0.946	0.053	0.826	0.920
1000	0.050	0.050	0.052	0.408	0.985	0.054	0.823	0.936

**Table 2:** Validation of Theorem 2. Rejection rate for hypotheses $H_{0}^{*}$ and $H_{A}^{*}$ of level α=0.05 in exponentially weighted bivariate HAR models of order p=3; **h** = (1,5,22), with decay rates $\lambda_{j}\in \left\{0.2,0.4,0.5,0.8\right\}$, j=1,2; $\left(\beta_{10},c_{11},c_{12}\right)=\left(1.0,0.12,0.01\right)$, $\left(\beta_{20},c_{21},c_{22}\right)=\left(1.0,0.07,0.01\right)$; replication number =1000.

*n*	$\lambda_{1}=0.2$		$\lambda_{1}=0.4$		$\lambda_{1}=0.5$	
	$\lambda_{2}=0.2$	$\lambda_{2}=0.8$	$\lambda_{2}=0.4$	$\lambda_{2}=0.8$	$\lambda_{2}=0.5$	$\lambda_{2}=0.8$
	$H_{0}^{*}$	$H_{A}^{*}$	$H_{0}^{*}$	$H_{A}^{*}$	$H_{0}^{*}$	$H_{A}^{*}$
100	0.027	0.301	0.021	0.340	0.028	0.326
200	0.033	0.288	0.032	0.299	0.043	0.274
500	0.050	0.284	0.045	0.266	0.048	0.258
1000	0.048	0.285	0.051	0.265	0.049	0.270

It is shown from Table 1 that Case I favors two of the null hypotheses in Theorem 1; i.e., the models are exponentially weighted with small rejection numbers of the null hypothesis. Moreover, Table 2 depicts reasonable rejection rates of the test in Theorem 2 for a large sample size under both null and alternative hypotheses. Note that in Table 2, the null hypothesis $H_{0}^{*}$ indicates that both $\lambda_{1}$ and $\lambda_{2}$ are the same values; i.e., the common rate $\lambda =\lambda_{1}=\lambda_{2}\in \left\{0.2,0.4,0.5\right\}$, where small rejection rates are reported. The rejection rates in Table 1 and Table 2 tend to the nominal level α=0.05 as *n* increases under the null hypothesis.

Next, we observe the size and power properties of our proposed CUSUM test in Theorem 1. To assess the performance of the test, we use bivariate HAR models of orders p=3,6. We set h=(1,5,22), (1,7,14) if p=3, and h=(1,5,7,9,14,22), (1,7,14,19,22,25) if p=6. The sizes of the proposed test in the HAR(p,2) models with $\lambda_{1}\in \left\{0.5,0.8\right\},\lambda_{2}\in \left\{0.1,0.4\right\}$, are illustrated in Table 3. Most cases indicate very small values of type I errors, consistent with the size of the test. Also, the powers of the CUSUM test are displayed in Table 4, where we see comparatively reasonable power results.

In Table 5, estimates of the decay rates in (9) and (10) are computed to obtain sample means and standard errors. Cases I–III have the same parameters as those in Table 1 whereas Cases IV${}^{*}$–VI${}^{*}$ use common rates given as follows:

• Case IV${}^{*}$: Common rate $\lambda_{1}=\lambda_{2}=\lambda =0.1$.
• Case V${}^{*}$: Common rate $\lambda_{1}=\lambda_{2}=\lambda =0.5$.
• Case VI${}^{*}$: Common rate $\lambda_{1}=\lambda_{2}=\lambda =0.9$.

Table 5 reports that estimate results are consistent in the sample sizes, whereas for Cases IV${}^{*}$, V${}^{*}$ and VI${}^{*}$, estimates ${\hat{\lambda }}_{j}$ in (9) are used for $\lambda_{j}$, j=1,2, and estimates $\hat{\lambda }$ in (10) are used for λ. We notice that in the common rate cases, Equation (10) gives smaller standard errors of the estimates.

**Table 3:** Size of the CUSUM test in Theorem 1 for HAR(p,2), p=3,6; $\left(\beta_{10},c_{11},c_{12}\right)=\left(1.0,0.12,0.10\right)$, $\left(\beta_{20},c_{21},c_{22}\right)=\left(1.0,0.07,0.25\right)$; Replication number = 1000, α=0.05.

*p*	h	*n*	$\lambda_{1}=0.5$				$\lambda_{1}=0.8$			
			$\lambda_{2}=0.1$		$\lambda_{2}=0.4$		$\lambda_{2}=0.1$		$\lambda_{2}=0.4$	
			$H_{1,0}$	$H_{2,0}$	$H_{1,0}$	$H_{2,0}$	$H_{1,0}$	$H_{2,0}$	$H_{1,0}$	$H_{2,0}$
p=3	(1, 5, 22)	100	0.087	0.078	0.080	0.067	0.091	0.090	0.094	0.078
		200	0.071	0.071	0.074	0.059	0.071	0.075	0.061	0.061
		500	0.054	0.058	0.050	0.054	0.052	0.054	0.051	0.050
		1000	0.050	0.050	0.050	0.053	0.051	0.052	0.050	0.050
p=6	(1, 5, 7, 9, 14, 22)	100	0.098	0.089	0.108	0.078	0.076	0.065	0.054	0.053
		200	0.064	0.084	0.058	0.071	0.058	0.053	0.052	0.052
		500	0.054	0.076	0.049	0.069	0.051	0.056	0.051	0.050
		1000	0.049	0.055	0.045	0.058	0.047	0.056	0.050	0.051
p=3	(1, 7, 14)	100	0.112	0.091	0.105	0.081	0.099	0.093	0.095	0.076
		200	0.070	0.064	0.060	0.061	0.071	0.072	0.069	0.059
		500	0.054	0.051	0.052	0.053	0.052	0.056	0.050	0.053
		1000	0.050	0.051	0.052	0.053	0.050	0.050	0.050	0.052
p=6	(1, 7, 14, 19, 22, 25)	100	0.091	0.090	0.077	0.076	0.071	0.064	0.057	0.053
		200	0.076	0.076	0.073	0.069	0.061	0.053	0.056	0.050
		500	0.053	0.065	0.065	0.055	0.048	0.048	0.047	0.049
		1000	0.054	0.062	0.055	0.051	0.048	0.052	0.050	0.051

**Table 4:** Power of the CUSUM test in Theorem 1 for HAR(p,2), *p* = 3,6; Replication number = 1000, α=0.05. (Parameter values used in Power Models 1 & 2 are available upon request).

*n*	Power Model 1				Power Model 2			
	p=3		p=6		p=3		p=6	
	(1,5,22)		(1,5,7,9,14,22)		(1,7,14)		(1,7,14,19,22,25)	
	$H_{1,A}$	$H_{2,A}$	$H_{1,A}$	$H_{2,A}$	$H_{1,A}$	$H_{2,A}$	$H_{1,A}$	$H_{2,A}$
100	0.664	0.807	0.716	0.518	0.812	0.727	0.691	0.722
200	0.794	0.906	0.849	0.642	0.901	0.823	0.679	0.771
500	0.850	0.967	0.948	0.806	0.937	0.863	0.738	0.852
1000	0.907	0.987	0.976	0.841	0.948	0.887	0.802	0.952

Finally, we discuss the fitting problem of the exponentially weighted HAR models in (12) and compare with the conventional HAR model fitted by OLSEs. The simulated data in Figure 1 are used to compute the OLSEs ${\hat{\beta }}_{j,O}$ of coefficients and the estimates ${\hat{\lambda }}_{j}$ of the decay rates in (9). From the estimated rates ${\hat{\lambda }}_{j}$, the rate-adopted estimators (REs) ${\tilde{\beta }}_{j,\Lambda }$ of the coefficients are evaluated as a further step. To compare the fitted models by the OLSEs and REs, Table 6 presents some criteria such as the root mean square error (RMSE), mean absolute error (MAE), AIC, and BIC. In the case of the exponential weighted bivariate HAR models, the fitting parameter *m* is used with m=5,10,20,100. Because the conventional bivariate HAR model has 14 parameters whereas our proposed model has 8 parameters, the AIC and BIC of the latter are smaller values than those of the former. Intuitively, the small choice of *m* yields small errors in RMSEs and MAEs because the average ${\bar{\omega }}_{m,t}$ in (11) for interval [t−m,t+m] is closer to ${\tilde{\epsilon }}_{j,t,\Lambda }$ for smaller *m*, and thus this fact makes the error term $\varepsilon_{j,t}$ in (12) smaller. In the latter case, (12) is applied with m=5 for the bias adjustment. RMSE and MAE of the OLSE residuals in fitting the conventional HAR model are 0.9971, 0.8253 for j=1 and 0.9644, 0.7596 for j=2, and those of the RE residuals in fitting our proposed model are 0.9424, 0.7805 for j=1 and 0.9226, 0.7381 for j=2.

**Table 5:** Sample means and standard errors (s.e.) of estimates for the decay rates $\lambda_{1},\lambda_{2}$ of exponentially weighted HAR (3, 2) models, Replication number = 1000. Note that in common rate cases with *, Cases IV${}^{*}$, V${}^{*}$ and VI${}^{*}$, estimates ${\hat{\lambda }}_{j}$ in (9) are used for $\lambda_{j}$, j=1,2, while estimates $\hat{\lambda }$ in (10) are used for λ.

		n=500		n=1000		n=2000	
		**Sample Mean**	**(s.e.)**	**Sample Mean**	**(s.e.)**	**Sample Mean**	**(s.e.)**
*Case I*	$\lambda_{1}=0.5$	0.458	(0.038)	0.493	(0.017)	0.528	(0.008)
	$\lambda_{2}=0.4$	0.418	(0.022)	0.452	(0.012)	0.419	(0.005)
*Case II*	$\lambda_{1}=0.5$	0.764	(0.143)	0.625	(0.021)	0.505	(0.007)
	-		-		-		-
*Case III*	-		-		-		-
	$\lambda_{2}=0.4$	0.497	(0.034)	0.445	(0.014)	0.413	(0.006)
Case IV${}^{*}$	$\lambda_{1}=0.1$	0.188	(0.037)	0.121	(0.019)	0.104	(0.011)
	$\lambda_{2}=0.1$	−0.022	(0.164)	0.101	(0.011)	0.103	(0.007)
	λ=0.1	0.118	(0.014)	0.084	(0.009)	0.095	(0.006)
Case V${}^{*}$	$\lambda_{1}=0.5$	0.684	(0.044)	0.579	(0.019)	0.525	(0.012)
	$\lambda_{2}=0.5$	0.556	(0.019)	0.526	(0.012)	0.518	(0.008)
	λ=0.5	0.507	(0.015)	0.511	(0.010)	0.506	(0.007)
Case VI${}^{*}$	$\lambda_{1}=0.9$	0.768	(0.652)	0.934	(0.021)	0.921	(0.014)
	$\lambda_{2}=0.9$	0.983	(0.021)	0.928	(0.014)	0.922	(0.010)
	λ=0.9	0.930	(0.018)	0.888	(0.011)	0.900	(0.008)

**Table 6:** Comparison of exponential weighted HAR(3,2) fitting models from the simulated data in Figure 1.

Model	Fitting	j=1				j=2			
	Parameter *m*	RMSE	MAE	AIC	BIC	RMSE	MAE	AIC	BIC
Conventional HAR	-	0.9971	0.8253	1098.49	1153.57	0.9644	0.7596	1073.38	1128.47
Exp. HAR	m=5	0.9424	0.7805	1043.88	1075.36	0.9226	0.7381	1027.80	1059.28
Exp. HAR	m=10	0.9898	0.8147	1080.84	1112.32	0.9443	0.7535	1045.34	1076.82
Exp. HAR	m=20	0.9949	0.8180	1084.83	1116.31	0.9709	0.7621	1066.24	1097.71
Exp. HAR	m=100	1.0105	0.8334	1096.23	1127.70	0.9827	0.7745	1075.59	1107.07

Furthermore, to elaborate more on the comparison with the conventional HAR model, the efficiency of the proposed model vs. the conventional one, is computed by using two metrics of RMSE and MAE: The Exp. HAR Model Efficiency, relative to the benchmark HAR model, is defined by

$$\mathrm{Effi}\_{}_{RMSE}=\left(\frac{RMSE_{0}-RMSE_{1}}{RMSE_{1}}\right)\times 100,\;\;\;\;\mathrm{Effi}\_{}_{MAE}=\left(\frac{MAE_{0}-MAE_{1}}{MAE_{1}}\right)\times 100,$$

where *RMSE*${}_{0}$ and *MAE*${}_{0}$ are *RMSE*, *MAE* of the conventional HAR model, respectively and *RMSE*${}_{1}$ and *MAE*${}_{1}$ are those of the exponentially weighted HAR model. Table 7 displays the Exp. HAR model efficiency in the first case of $\left(\lambda_{1},\lambda_{2}\right)$ of Table 3. We see that all values are positive with highest value 7.0817 in percentage, which means that the proposed model with the RE fitting improves the conventional HAR model with respect to residual errors.

**Table 7:** Comparison with conventional HAR model by computing Exp. HAR model efficiency, defined by Effi${}_{\_RMSE}=$$100\times \left(RMSE_{0}-RMSE_{1}\right)/RMSE_{1}$, Effi${}_{\_MAE}=100\times \left(MAE_{0}-MAE_{1}\right)/MAE_{1}$ of exponential weighted HAR model, where RMSE${}_{0}$, MAE${}_{0}$ are the root mean square error (RMSE), and mean absolute error (MAE) of the conventional HAR model, respectively; and RMSE${}_{1}$, MAE${}_{1}$ are those of Exp. HAR model.

	n=500				n=1000			
Rates	*Effi* ${}_{\_\mathrm{RMSE}}$		*Effi* ${}_{\_\mathrm{MAE}}$		*Effi* ${}_{\_\mathrm{RMSE}}$		*Effi* ${}_{\_\mathrm{MAE}}$	
($\lambda_{1},\lambda_{2}$)	j=1	j=2	j=1	j=2	j=1	j=2	j=1	j=2
(0.5, 0.1)	4.1635	6.3361	5.2112	4.3094	4.8475	4.9433	7.0817	6.9621
(0.5, 0.4)	5.1794	6.8979	5.0989	2.8984	5.8510	5.4411	5.3964	5.4328
(0.8, 0.1)	5.9158	4.4523	4.5951	5.1982	5.3078	6.3053	4.7309	6.9481
(0.8, 0.4)	5.7379	3.6783	6.3833	3.7651	5.1028	4.4709	5.7066	6.0533

The HAR model has high applicability in the financial market. In particular, it is very powerful for the realized volatility forecasting [1,17,18,29]. However, besides volatility forecasting, as a theoretically linear AR model, it has many applications to various time series data as in [12,13,14,42]. The exponentially weighted multivariate HAR model, which is one of the special cases of HAR models, is suitable for joint data with strong cross-correlation. The decay rate of the model plays a key role in the common structure of the joint data with strong correlation. In economics and finance, there are many strongly correlated time series data that are important for policy decisions to improve the global economy and human society. For example, stock prices in the same category tend to have the same pattern. Stock price modellings are known to be based on efficient market hypotheses (EMH), according to which only relevant information on the stock is its current values. The proposed model may be proper to the stock price modelling because the current value and the current averages (with exponentially decaying coefficients) are used as regressor variables in the model. The following section will address empirical analysis of the joint data of strongly correlated stock prices to confirm the intuition of the proposed model for the stock prices.

## 4. Empirical Analysis

**Figure 7:**
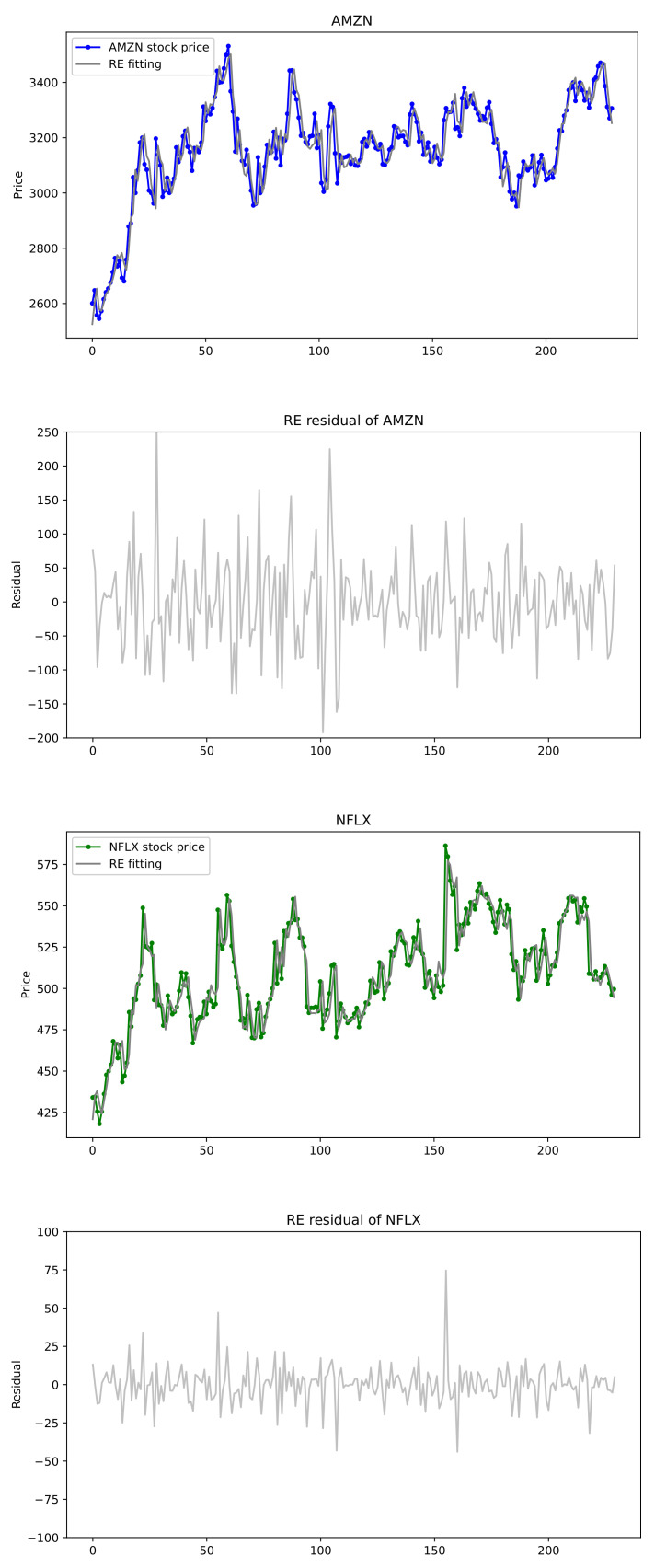
(AMZN, NFLX) stock prices and exponentially weighted HAR(3,2) models fitted by REs with their residuals.

**Figure 8:**
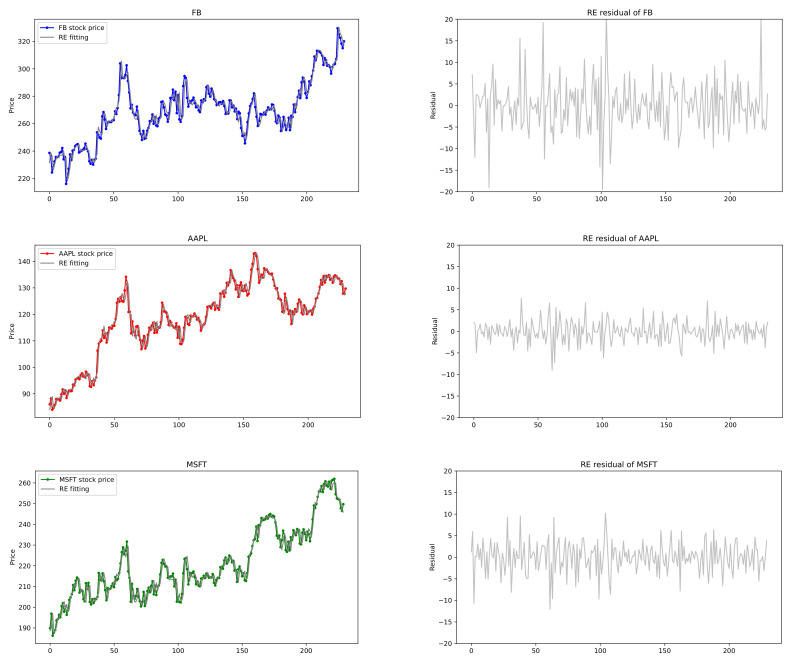
(FB, AAPL, MSFT) stock prices and exponentially weighted HAR(3,3) models fitted by REs with their residuals.

In this section, we provide empirical examples of U.S. stock prices that are applied to the exponentially weighted multivariate HAR models. Note that realized volatility does not fit for our model, but the stock price itself may be suitable for the proposed model with exponential decay coefficients. To this end, we choose some datasets of U.S. stock prices and conduct our proposed CUSUM tests. For a bivariate joint data (q=2), stock prices of Amazon.com Inc. (AMZN) (Seattle, WA, USA) and Netflix Inc. (NFLX) (Los Gatos, CA, USA), and for a triple joint data (q=3), those of Apple Inc. (AAPL) (Cupertino, CA, USA), Microsoft Corporation (MSFT) (Redmond, WA, USA) and Facebook Inc. (FB) (Menlo Park, CA, USA) are selected from 7 May 2020 to 6 May 2021. In the analysis, closing price is chosen because it reflects all the activities in a trading day. Plots of these stock prices are shown in Figure 7 and Figure 8, where we see that the time series data reveal somewhat similar patterns for the pair (AMZN, NFLX) and for the triple (FB, AAPL, MSFT).

First we adopt bivariate HAR(3,2) models for pairs of (AMZN, NFLX), (AAPL, MSFT), (FB, AAPL) and (FB, MSFT) for q=2, respectively, and second a multivariate HAR(3,3) model for three datasets (FB, AAPL, MSFT) for q=3. Order p=3 and lag h=(1,5,22) are used. Results of tests and estimates as well as correlation coefficients are reported in Table 8, where suprema of test statistics and decay rate estimates are computed by Theorem 1 and Equation (9), respectively. More specifically, conducting the CUSUM test in Theorem 1 to detect the presence of the exponential decay rates, test statistics are evaluated as follows. In the case of (AMZN, NFLX) for detecting the existence of $\lambda_{AMZN}$ and $\lambda_{NFLX}$, the CUSUM test statistics are computed as 0.3986 for AMZN and 0.3489 for NFLX. In the case of (AAPL, MSFT), the CUSUM test statistics are computed as 0.7508 for AAPL and 0.4979 for MSFT. These values imply that the null hypothesis is not rejected because the critical values of the standard Brownian bridge are 1.224 of level α=0.1 and 1.358 of level α=0.05. On the other hand, as the test in Theorem 2 for a common rate is conducted, the test statistics are evaluated as values greater than 2, which rejects the null hypothesis with the common rate.

Now comparisons with the conventional HAR models are presented for the two pairs (AMZN, NFLX), (AAPL, MSFT), and for the triple (FB, AAPL, MSFT). We compare performances for these datasets applied to univariate HAR model, (conventional) multivariate HAR model and exponentially weighted multivariate HAR (Exp. HAR) model. For the conventional HAR models, two estimation methods of OLSE and LASSO used in [43] are adopted. LASSO estimates are computed by LassoLarsCV in sklearn.linear_model with Python version 3.8.6. For the Exp. HAR model, the RE is computed. In Table 9, for two joint datasets (q=2), the conventional HAR(3,2) model has 14 parameters, whereas the exponential weighted HAR(3,2) model has 8 parameters. Each univariate HAR model has 4 parameters, so the total number is 8. Table 9 reports the comparison results of the HAR(3,2) models. The CUSUM test favors the existence of exponential decay rates, and thus the exponential bivariate HAR(3,2) is fitted with the rate estimates. The decay rates for AMZN and NFLX are estimated as $\left(\lambda_{AMZN},\lambda_{NFLX}\right)=\left(0.2983,0.1144\right)$ whereas those for AAPL and MSFT are $\left(\lambda_{AAPL},\lambda_{MSFT}\right)=\left(0.02175,-0.01483\right)$, which are presented in Table 8. Four measures of RMSE, MAE, AIC, and BIC are compared in the three models via OLSE, LASSO, and RE. In Table 9, the best values are displayed in bold. The exponential bivariate HAR(3,2) model has the best performance on RMSE and MAE, whereas the univariate HAR model with LASSO has the best performance on the AIC and BIC. Figure 7 depicts the actual data of stock prices of (AMZN, NFLX) and the fitted Exp. HAR(3,2) model by the REs along with their residuals. It can be seen that the stock prices of (AMZN, NFLX) are well matched to the fitted model.

**Table 8:** Results of tests and estimates for (MZN, NFLX) and (FB, AAPL, MSFT) in exponentially weighted HAR(3,q) models, q=2,3.

	Correlation	*q*	Test Statistics	Rate Estimates
	Coefficient		$\sup_{0\leq z\leq 1}\left|{\hat{T}}_{j,n}\left(z\right)\right|,\;\left(j=1,\cdots ,q\right)$	${\hat{\lambda }}_{j},\;\left(j=1,\cdots ,q\right)$
(AMZN, NFLX)	0.8268	2	(0.3986, 0.3489)	(0.2983,0.1144)
(AAPL, MSFT)	0.8396	2	(0.7508, 0.4979)	(0.02175,−0.01483)
(FB, AAPL)	0.8185	2	(0.4644, 0.7925)	(−006340,0.03158)
(FB, MSFT)	0.8203	2	(0.4549, 0.4794)	(0.01255, 0.01322)
(FB, AAPL, MSFT)	-	3	(0.4889, 0.7424, 0.4762)	(−0.03302,0.03097,0.02733)

**Table 9:** Comparison of univariate HAR, bivariate HAR and exponential weighted bivariate HAR models for (AMZN, NFLX), (AAPL, MSFT) stock prices from 7 May 2020 to 6 May 2021; p=3, h=(1,5,22); ${}^{*}\left(\lambda_{AMZN},\lambda_{NFLX}\right)=\left(0.2983,0.1144\right)$, ${}^{*}\left(\lambda_{AAPL},\lambda_{MSFT}\right)=\left(0.02175,-0.01483\right)$.

		Univariate HAR(3)		Bivariate HAR(3,2)		Exp. Bi. HAR(3,2)${}^{*}$
Total # of Parameters		8		14		8
	Estimator	OLSE	LASSO	OLSE	LASSO	RE
AMZN	RMSE	61.6182	62.9610	61.5780	62.9610	**61.2005**
	MAE	47.1019	47.6977	47.1447	47.6977	**46.2293**
	AIC	2556.35	**2321.29**	2576.05	2341.28	2561.21
	BIC	2570.10	**2334.64**	2624.18	2388.01	2588.71
NFLX	RMSE	13.3562	13.6602	13.3174	13.8798	**12.3834**
	MAE	9.0379	9.2866	9.0565	9.9867	**8.4594**
	AIC	1853.02	**1685.89**	1871.68	1712.52	1826.23
	BIC	1866.77	**1699.25**	1919.82	1759.25	1853.73
AAPL	RMSE	2.6255	2.6866	2.6061	2.6609	**2.4899**
	MAE	1.9478	1.9877	1.9346	1.9709	**1.8520**
	AIC	1104.74	**1009.34**	1121.31	1025.34	1088.24
	BIC	1118.48	**1022.69**	1269.45	1072.06	1115.75
MSFT	RMSE	3.8234	3.8979	3.8208	3.8975	**3.5746**
	MAE	2.9249	2.9625	2.9315	2.9633	**2.7488**
	AIC	1277.63	**1164.14**	1297.33	1184.10	1254.68
	BIC	1291.39	**1177.49**	1345.46	1230.83	1282.19

Table 10 reports the performances of the HAR(3,3) models for (FB, AAPL, MSFT). The conventional HAR(3,3) model has 30 parameters, whereas the exponential weighted HAR(3,3) model has 15 parameters. The decay rate estimates of (FB, AAPL, MSFT) are $\left(\lambda_{FB},\lambda_{AAPL},\lambda_{MSFT}\right)=\left(-0.03302,0.03097,0.02733\right)$, from which Exp. HAR(3,3) models are fitted in Figure 8. As seen in Table 10, our proposed model performs better than others with respect to the RMSE, MAE, and AIC whereas the univariate HAR model with LASSO has good performance on BIC, which are indicated by bold numbers in Table 10. Consequently, the proposed model not only has fewer parameters than the conventional HAR models, but also yields the best performance on the loss errors such as RMSE and MAE. The exponentially weighted HAR model with decay rates is suitable for the stock prices of joint financial assets with strong cross-correlation, rather than the volatility, in the stock market.

## 5. Concluding Remark

This work presents the exponentially weighted multivariate HAR models with exponentially decaying coefficients. The models represent very well two main features: long memory and strong cross-correlation, of financial market data. The common structure of multivariate data, which has such features, can be expressed by the existence of the decay rates of the coefficients in the model. For detecting the existence of the decay rates in the multivariate HAR models, CUSUM-based tests are established in two stages. The first is whether the multivariate HAR model has an exponential decay rate for each asset. The second is whether the model has a common rate for all assets. To test the presence of the rates, difference series are generated from two types of residuals and the change-points of its mean or variance are detected from the pseudo-time series, but not the raw data. The null limiting distributions of the test statistics are derived to be the standard Brownian bridge and are used in providing the asymptotic critical values for rejection of the hypothesis. Easy-to-implement estimators of the decay rates are computed. A Monte Carlo simulation study verifies the proposed tests by illustrating the related series and evaluating finite sample performance of size and power of the tests. Empirical examples show the usefulness of our proposed models in the stock market, especially stock price, but not volatility. Fewer parameters and smaller residual errors in our models are demonstrated.

Let us consider four aspects: (i) decreased number of parameters; (ii) smaller model-fitting errors; (iii) representation of common structure with decay rates; and (iv) a suitable model for stock price movement. These are the main advantages of the exponentially weighted multivariate HAR models. These advantages will help to practically provide more efficient models with smaller errors of predictions in financial time series modelling. In economics and finance, many strongly correlated data of joint assets play a crucial role in policymaking on economic and social regulations. The multivariate feature of our proposed model could be useful to improve forecasting accuracy of the financial assets and, thus it is possible to fine-tune policymaking on such asset classes.

The HAR model has very high applicability in the financial market. An extension of our proposed model can be useful to joint mutivariate data with strong correlation and long memory. In particular, like [12,13,14,15], an extended model incorporating exogenous variables such as an associated-uncertainty index would be a good prediction model to assess the high forecasting gain. For example, joint datasets, like gold and silver, oil and exchange rate, or several stock prices in the same sector, are affected together by global issues like COVID-19 and the Ukrainian War in the current decade. Such a uncertainty-related index can be added to the model as regressors and the extended multivariate HAR model is expected to give a good performance.

Also, an extension of the proposed model can be established as a dynamic time series model that is more applicable to real-world market data, for example, with time-varying variance, non-Gaussianity or heavy-tailed distribution. Recently, Ref. [18] analyzed a multivariate HAR-RV model with GARCH errors, for which a weighting scheme based on the conditional variances of the errors is used to construct the weighted least squares estimates. An extension of this work can be linked to heteroscedasticity. Exponentially weighted multivariate HAR models with time-varying variances such as GARCH errors or ARCH without intercept (see Ref. [44]) would be interesting topics. Reference [44] proposed a double AR model without an intercept (DARWIN model) as a modification of an AR-ARCH model as follows: $y_{t}=\phi y_{t-1}+\eta_{t}\sqrt{\alpha y_{t-1}^{2}}$ where $\phi \in R,\;\alpha >0,\;\{\eta_{t}\}$ is i.i.d. with zero mean and unit variance, and independent of $\left\{y_{j}:j<t\right\}$. The DARWIN model is nonstationary and heteroscedastic regardless of the sign of Lyapunov exponent, and hence it provides us a new way to model the nonstationary heteroscedastic time series. Analysis on a nonstationary, exponentially weighted HAR model combined with the DARWIN model will be an interesting topic in modelling heteroscedastic time series data. In the exponentially weighted multivariate HAR model with the DARWIN errors, statistical methods for detecting and estimating the decay rates along with the DARWIN parameter estimation will be a challenging study.

## Figures and Tables

**Table 10:** Comparison of univariate HAR, multivariate HAR, and exponential weighted multivariate HAR models for (FB, AAPL, MSFT) stock prices from 7 May 2020 to 6 May 2021; p=3, h=(1,5,22); ${}^{*}\left(\lambda_{FB},\lambda_{AAPL},\lambda_{MSFT}\right)=\left(-0.03302,0.03097,0.02733\right)$.

		Univariate HAR(3)		Multivariate HAR(3,3)		Exp. Multi. HAR(3,3) ${}^{*}$
Total # of Parameters		12		30		15
	Estimator	OLSE	LASSO	OLSE	LASSO	RE
FB	RMSE	6.1757	6.1953	6.0512	6.1571	**5.7108**
	MAE	4.5110	4.5899	4.5443	4.5703	**4.2440**
	AIC	1498.20	1356.83	1540.83	1406.19	**1345.09**
	BIC	1511.95	**1370.18**	1643.97	1506.32	1395.15
AAPL	RMSE	2.6255	2.6866	2.5978	2.6884	**2.5393**
	MAE	1.9478	1.9877	1.9334	1.9831	**1.8996**
	AIC	1104.74	1009.34	1151.86	1061.76	**1007.94**
	BIC	1118.48	**1022.69**	1255.01	1161.63	1058.00
MSFT	RMSE	3.8234	3.8979	3.8057	3.9024	**3.6608**
	MAE	2.9249	2.9625	2.9316	2.9445	**2.8092**
	AIC	1277.63	1164.14	1327.49	1216.41	**1160.11**
	BIC	1291.39	**1177.49**	1430.64	1316.53	1210.17

## Appendix A. Proofs

**Proof** **of** **Theorem 1.** From (6), we write $D_{j,t}={\hat{\Delta }}_{j}^{⊤}{\tilde{W}}_{j,t-1}$ for simplicity, where

$${\hat{\Delta }}_{j}={\left({\hat{\lambda }}_{j,2}-{\hat{\lambda }}_{j,1}^{2},\;\cdots ,\;{\hat{\lambda }}_{j,p-1}-{\hat{\lambda }}_{j,1}^{p-1}\right)}^{⊤},\;\;\;{\tilde{W}}_{j,t-1}={\left({\hat{W}}_{j,t-1}^{\left(3\right)},\cdots ,{\hat{W}}_{j,t-1}^{\left(p\right)}\right)}^{⊤}.$$

We fix *j* and first prove

(A1) $$S_{j,n}=\frac{1}{{\hat{\sigma }}_{j,D}\sqrt{n}}\sum_{t=1}^{n}D_{j,t}\to^{d}N\left(0,1\right).$$

Let $\sigma_{j,D}^{2}=\lim_{n\to \infty }{\hat{\sigma }}_{j,D}^{2}$ in probability, and let

$$X_{n,t}=\frac{1}{\sigma_{j,D}\sqrt{n}}D_{j,t}=\frac{1}{\sigma_{j,D}\sqrt{n}}{\hat{\Delta }}_{j}^{⊤}{\tilde{W}}_{j,t-1}.$$

$\left\{X_{n,t}:t=1,2,\cdots ,n;\;n=1,2,\cdots \right\}$ is a triangular array and it depends on *j*, but the subscript is omitted for notational simplicity. It is clear that

(A2) $$\sum_{t=1}^{n}X_{n,t}^{2}\to^{p}1.$$

Note that for each k,j∈{1,⋯,p}, ${\hat{\lambda }}_{j,k}-{\hat{\lambda }}_{j,1}^{k}=O_{p}\left(1/\sqrt{n}\right)$ because the OLSE ${\hat{\lambda }}_{j,k}$ of $\lambda_{j,k}$ satisfies the asymptotic normality of $\sqrt{n}\left({\hat{\lambda }}_{j,k}-\lambda_{j,k}\right)$ with asymptotic mean zero. Moreover, we may have the asymptotic normality: $\sqrt{n}\left({\hat{\lambda }}_{j,k}-{\hat{\lambda }}_{j,1}^{k}\right)\to^{d}N\left(0,\upsilon_{k}^{2}\right)$ for some $\upsilon_{k}^{2}>0$. From this, and from the boundedness of ${\hat{W}}_{j,t-1}^{\left(k\right)}$ under condition $E|Y_{j,t}{|}^{2+\delta }<\infty$, we have

(A3) $$E(\max_{t}|X_{n,t}|)\to 0.$$

For (A1) we prove $S_{n}\equiv S_{j,n}=\sum_{t=1}^{n}X_{n,t}\to^{d}N\left(0,1\right)$. It suffices to show

(A4) $$E\left[\exp\left(\iota xS_{n}\right)\right]\to \exp\left(-\frac{x^{2}}{2}\right)$$

where $\iota =\sqrt{-1}$ and x∈R. Let $Z_{n,1}=X_{n,1}$ and $Z_{n,t}=X_{n,t}I\left(\sum_{s=1}^{t-1}X_{n,s}^{2}\leq 2\right)$ for 2≤t≤n, where I(·) is the indication function. Let $J=\inf\{t:\sum_{s=1}^{t}X_{n,s}^{2}>2\}\wedge n$. Then

(A5) $$P\left(X_{n,t}\neq Z_{n,t}\;\mathrm{for}\;\mathrm{some}\;t\leq n\right)=P\left(J\leq n-1\right)\leq P\left(\sum_{s=1}^{n}X_{n,s}^{2}>2\right)\to 0$$

by (A2). Now we use the following expansion: $\exp\left(\iota x\right)=\left(1+\iota x\right)\exp\left(-\frac{x^{2}}{2}+R\left(x\right)\right)$ where R(x) is some function with $\left|R\left(x\right)\right|\leq {\left|x\right|}^{3}$. Let

$$V_{n}=\prod_{t=1}^{n}\left(1+\iota xZ_{n,t}\right)\;\;\mathrm{and}\;\;U_{n}=\exp\left(-\frac{x^{2}}{2}\sum_{t=1}^{n}Z_{n,t}^{2}+\sum_{t=1}^{n}R\left(xZ_{n,t}\right)\right).$$

Note that $V_{n}U_{n}=\exp\left(\iota xS_{n}\right)$ and $U_{n}\to^{p}\exp\left(-\frac{x^{2}}{2}\right)=:a$ by (A2) and (A5). Because $V_{n}U_{n}=V_{n}\left(U_{n}-a\right)+aV_{n}$, we may show that $|V_{n}|$ is uniformly integrable and $E(V_{n})\to 1$. We observe

$$|V_{n}|=\prod_{t=1}^{n}\left|\left(1+\iota xZ_{n,t}\right)\right|=\prod_{t=1}^{J-1}{\left(1+x^{2}X_{n,t}^{2}\right)}^{1/2}{\left(1+x^{2}X_{n,J}^{2}\right)}^{1/2}$$

$$\leq \exp\left(\frac{x^{2}}{2}\sum_{t=1}^{J-1}X_{n,t}^{2}\right)\left(1+|x|\right|X_{n,J}\left|\right)\leq \exp\left(x^{2}\right)\left(1+|x\right|\max_{t}|X_{n,t}\left|\right),$$

where the inequality ${\left|\left(1+\iota xz\right)\right|}^{2}=\left(1+x^{2}z^{2}\right)\leq \exp\left(x^{2}z^{2}\right)$ is used. Thus, by (A3), $V_{n}$ is uniformly integrable. Finally we show that $E(V_{n})\to 1$. Let

$$F_{n,t}=\sigma \left\{{\hat{\Delta }}_{j},{\tilde{W}}_{j,s}:-\infty <s\leq t,j=1,\cdots ,q\right\}$$

with $F_{n,t-1}\subset F_{n,t}$. Set $I_{t-1}:=I\left\{\sum_{s=1}^{t-1}X_{n,s}^{2}\leq 2\right\}$. We have $E(V_{n})=$

$$E\left[\prod_{t=1}^{n}\left(1+\iota xX_{n,t}I\left\{\sum_{s=1}^{t-1}X_{n,s}^{2}\leq 2\right\}\right)\right]=E\left[E\left[\prod_{t=1}^{n}\left(1+\frac{\iota x}{\sigma_{j,D}\sqrt{n}}{\hat{\Delta }}_{j}^{⊤}{\tilde{W}}_{j,t-1}I_{t-1}\right|F_{n,n-1}\right]\right]$$

(A6) $$=E\left[\prod_{t=1}^{n-1}\left(1+\frac{\iota x}{\sigma_{j,D}\sqrt{n}}{\hat{\Delta }}_{j}^{⊤}{\tilde{W}}_{j,t-1}I_{t-1}E\left[1+\frac{\iota x}{\sigma_{j,D}\sqrt{n}}{\hat{\Delta }}_{j}^{⊤}{\tilde{W}}_{j,n-1}I_{n-1}\right|F_{n,n-1}\right]\right].$$

We also observe

$$E\left[\left.1+\frac{\iota x}{\sigma_{j,D}\sqrt{n}}{\hat{\Delta }}_{j}^{⊤}{\tilde{W}}_{j,n-1}I_{n-1}\right|F_{n,n-1}\right]=E\left[\left.1+\frac{\iota x}{\sigma_{j,D}\sqrt{n}}\sum_{k=2}^{p-1}\left({\hat{\lambda }}_{j,k}-{\hat{\lambda }}_{j,1}^{k}\right){\hat{W}}_{j,n-1}^{\left(k+1\right)}I_{n-1}\right|F_{n,n-1}\right]$$

$$=\;\;\left.1+\frac{\iota x}{\sigma_{j,D}\sqrt{n}}\sum_{k=2}^{p-1}E\left[\left({\hat{\lambda }}_{j,k}-{\hat{\lambda }}_{j,1}^{k}\right){\hat{W}}_{j,n-1}^{\left(k+1\right)}I_{n-1}\right|F_{n,n-1}\right]\;\;\;\;\;$$

(A7) $$=\;\;\left.1+\frac{\iota x}{\sigma_{j,D}\sqrt{n}}\sum_{k=2}^{p-1}E\left[\frac{1}{\sqrt{n}}\left(\Gamma_{k}+o_{p}\left(1\right)\right){\hat{W}}_{j,n-1}^{\left(k+1\right)}I_{n-1}\right|F_{n,n-1}\right]$$

where $\Gamma_{k}$ is a normal random variable, (with mean $E[\Gamma_{k}]=0$), which is independent of ${\hat{W}}_{j,n-1}^{\left(k+1\right)}$. The expectation in (A7) can be written as

$$\frac{1}{\sqrt{n}}E\left[\left.\Gamma_{k}{\hat{W}}_{j,n-1}^{\left(k+1\right)}I_{n-1}\right|F_{n,n-1}\right]+o_{p}\left(1/\sqrt{n}\right)=\;\;\;\;\;\;\;\;\;\;\;\;\;\;\;\;\;\;\;\;\;\;\;\;$$

$$\frac{1}{\sqrt{n}}E[\left.\Gamma_{k}]E\left[{\hat{W}}_{j,n-1}^{\left(k+1\right)}I_{n-1}\right|F_{n,n-1}\right]+o_{p}\left(1/\sqrt{n}\right)=o_{p}\left(1/\sqrt{n}\right).$$

Thus, the last expression in (A7) is $1+o_{p}\left(1/n\right)$. Proceed this argument of successive conditioning to (A6) to obtain that $E\left[V_{n}\right]=E\left[\prod_{t=1}^{n}\left(1+o_{p}\left(1/n\right)\right]\right]=\exp\left(o_{p}\left(1\right)\right)\to 1.$ Therefore,

$$E\left[\exp\left(\iota xS_{n}\right)\right]=E\left[V_{n}U_{n}\right]=E\left[V_{n}\left(U_{n}-a\right)\right]+aE\left[V_{n}\right]\to a:=\exp\left(-\frac{x^{2}}{2}\right),$$

which is (A4), and we complete the asymptotic normality of $\frac{1}{\sigma_{j,D}\sqrt{n}}\sum_{t=1}^{n}D_{j,t}\to^{d}N\left(0,1\right)$. Because $\sigma_{j,D}^{2}=\lim_{n\to \infty }{\hat{\sigma }}_{j,D}^{2}$, (A1) holds. Similarly, we can show that $S_{\left[nz\right]}:=\sum_{t=1}^{\left[nz\right]}X_{n,t}$ converges to the Brownian motion B(z). It follows that the desired result in Theorem 1 is obtained. □

**Proof** **of** **Theorem 2.** The proof is similar to that of Theorem 1. A key difference between proofs of Theorems 1 and 2 is in the following: Let $X_{n,t}^{*}=\frac{1}{\sigma_{D}^{*}\sqrt{n}}\sum_{j=1}^{q}\left(d_{j,t}^{2}-{\hat{\sigma }}_{j,d}^{2}\right)$ where $\sigma_{D}^{*2}=\lim_{n\to \infty }{\hat{\sigma }}_{D}^{*2}$ in probability, and let $S_{n}^{*}=\sum_{t=1}^{n}X_{n,t}^{*}$. By Theorem 4 of [41], we have the asymptotic normality of $\sqrt{n}\left({\hat{\lambda }}_{j}-\lambda \right)$ with asymptotic mean zero for all j=1,2,⋯,q under the null hypothesis with common rate λ. Thus ${\hat{\epsilon }}_{j,t}^{*}-\epsilon_{t}^{*}\left(j\right)=O_{p}\left(1/\sqrt{n}\right)$ as well as $d_{j,t}:={\hat{\epsilon }}_{j,t}^{*\;2}-\epsilon_{t}^{*}{\left(j\right)}^{2}=O_{p}\left(1/\sqrt{n}\right)$ for all *j*. So $\max_{t}\left|X_{n,t}^{*}\right|=o_{p}\left(1/n\right)\to^{p}0$. Instead of $X_{n,t}$ and $S_{n}$ in the proof of Theorem 1, we replace by $X_{n,t}^{*}$ and $S_{n}^{*}$ to obtain the same results, along with $V_{n}^{*}:=\prod_{t=1}^{n}\left(1+\iota xZ_{n,t}^{*}\right)$ and $E[V_{n}^{*}]\to 1$, where $Z_{n,t}^{*}$ is given in the same way with $X_{n,t}^{*}$. Asymptotic normality of $S_{n}^{2}$ holds as well as the desired limiting distribution in Theorem 2 holds. □
